# The Glypican-1/HGF/C-Met and Glypican-1/VEGF/VEGFR2 Ternary Complexes Regulate Hair Follicle Angiogenesis

**DOI:** 10.3389/fcell.2021.781172

**Published:** 2021-12-08

**Authors:** Charlie Colin-Pierre, Nicolas Berthélémy, Nicolas Belloy, Louis Danoux, Vincent Bardey, Romain Rivet, Solène Mine, Christine Jeanmaire, François-Xavier Maquart, Laurent Ramont, Stéphane Brézillon

**Affiliations:** ^1^ Université de Reims Champagne-Ardenne, SFR CAP-Santé (FED 4231), Laboratoire de Biochimie Médicale et Biologie Moléculaire, Reims, France; ^2^ CNRS UMR 7369, Matrice Extracellulaire et Dynamique Cellulaire-MEDyC, Reims, France; ^3^ BASF Beauty Care Solutions France SAS, Pulnoy, France; ^4^ P3M, Multiscale Molecular Modeling Platform, Université de Reims Champagne-Ardenne, Reims, France; ^5^ CHU de Reims, Service Biochimie-Pharmacologie-Toxicologie, Reims, France

**Keywords:** glypican 1, hair follicle angiogenesis, KORS, HDMEC, HGF

## Abstract

The hair renewal involves changes in the morphology of the hair follicle and its micro-vascularization. In alopecia, the hair cycle is accelerated, resulting in the formation of thinner and shorter hair. In addition, alopecia is associated with a decrease in the micro-vascularization of the hair follicles. In this study, the role of glypicans (GPCs) was analyzed in the regulation of the angiogenesis of human dermal microvascular endothelial cells (HDMEC). The analysis of glypican gene expression showed that GPC1 is the major glypican expressed by human keratinocytes of outer root sheath (KORS), human hair follicle dermal papilla cells (HHFDPC) and HDMEC. KORS were demonstrated to secrete VEGF and HGF. The HDMEC pseudotube formation was induced by KORS conditioned media (KORS_CM_). It was totally abrogated after GPC1 siRNA transfection of HDMEC. Moreover, when cleaved by phospholipase C (PLC), GPC1 promotes the proliferation of HDMEC. Finally, GPC1 was shown to interact directly with VEGFR2 or c-Met to regulate angiogenesis induced by the activation of these receptors. Altogether, these results showed that GPC1 is a key regulator of microvascular endothelial cell angiogenesis induced by VEGF and HGF secreted by KORS. Thus, GPC1 might constitute an interesting target to tackle alopecia in dermatology research.

## 1 Introduction

Forty to one hundred hairs are lost per day and constantly renewed. This renewal involves cyclic changes in the hair follicle (HF) (Myung and Ito, 2012; Sada and Tumbar, 2013). Each cycle is divided into three main stages: anagen, the growing phase of the hair; catagen, the HF size regression; and telogen, where the hair shaft remains anchored in the HF before being dislodged by the new growing hair.

A HF can be divided into three parts. The *infundibulum* is the part between the surface of the skin and the end of the sebaceous duct edged by stratified keratinized epithelium (Knutson, 1974). The isthmus extends from the end of the sebaceous duct to the bulb. It is made up of different concentric layers from the outside to the inside: the connective sheath, basal membrane, outer root sheath, inner root sheath, and hair shaft (Bernard, 2006). The bulb is composed of an epithelial part, the hair germinative matrix and a mesenchymal part, the dermal papilla. This latter consists of connective tissue containing papillary fibroblasts (Bouhanna and Reygagne, 1999). The HF is surrounded by capillaries emerging from a small set of capillaries in close contact with dermal papilla (Montagna and Ellis, 1957).

In case of alopecia, the cycles are shorter and new hairs become thinner and shorter (miniaturization), and they eventually stop growing back. Hair modification can have repercussions on the individual and his/her quality of life, including loss of self-esteem, social isolation, and depression (Hunt and McHale, 2005). Alopecia is also characterized by a decrease of the hair microvascularization and a recent study has shown that in the balding scalp, genes involved in HF vascularization are downregulated (Chew et al., 2016).

Hair is nourished by a set of capillaries in the middle of the dermal papilla. Other capillaries emerge, running up the wall of the follicle almost as far as the *infundibulum* (Montagna and Ellis, 1957). During HF cycles, the vascular network is rearranged: in the late anagen phase, the capillaries are distributed along the wall of the HF, whereas at the end of the catagen phase and in the telogen phase, the capillaries are essentially located at the level of the dermal papilla (Montagna and Ellis, 1957; Ellis and Moretti, 1959). The inhibition of perifollicular angiogenesis significantly delays hair shaft development (Mecklenburg et al., 2000). The HF diameter is correlated to vessel size and capillary surface area (Yano et al., 2001). The growth of a new and robust hair shaft requires fine-tuned regulation of the vascular network involving the proliferation and migration of endothelial hair cells (Carmeliet and Jain, 2011; Johnson and Wilgus, 2014), as well as fibroblasts, keratinocytes, and growth factors (Stenn et al., 1988). Vascular endothelial growth factor (VEGF) is the most studied growth factor in the vascularization of the HF (Yano et al., 2001; Gnann et al., 2013; Quan et al., 2017). It is produced by dermal papilla (Idali, 2016), keratinocytes of the outer root sheath (KORS), and endothelial cells (Yano et al., 2001).

A change in the distribution of heparan sulfate proteoglycans (HSPGs) during the hair growth cycle was previously described (Malgouries et al., 2008). HSPGs are known to regulate the proliferation, migration, and differentiation induced by growth factors (Karamanos et al., 2018). Moreover, HSPGs were previously described to regulate angiogenesis (Rapraeger et al., 2013; Kastana et al., 2019). There are two main families of membrane HSPGs. Syndecans are characterized by a transmembrane core protein to which sulfated glycosaminoglycan chains are attached (Häcker et al., 2005). Glypicans (GPCs) present a core protein to which sulfated glycosaminoglycan chains are covalently linked (heparan sulfate, dermatan sulfate or chondroitin sulfate). They are anchored to the cell membrane by a glycosylphosphatidylinositol (GPI) anchor (Häcker et al., 2005; Filmus et al., 2008). Both forms of GPCs (secreted or anchored), and the degree of sulfation of glycosaminoglycans play pivotal roles in their mechanism (Pye et al., 1998; Kreuger et al., 2004; Ayers et al., 2010). Depending on the sequestered growth factor, GPCs trigger a stimulatory (Yamamoto et al., 2013) or inhibitory effect (Dwivedi et al., 2013). The GPC family is composed of six different members. Among these proteins, GPC1 is composed of a 558 amino acid core protein with three heparan sulfate chains attached at S486, S488, and S490 (Awad et al., 2015). It has both a membrane-anchored form and a secreted soluble form (Filmus et al., 2008; Truong et al., 2016). Phospholipase C (PLC) and disintegrin and metalloproteinase 17 (ADAM17) are known to cleave the GPI anchor and to release soluble GPCs (Hereld et al., 1986; Kawahara et al., 2017). GPC1 has shown to enhance VEGF-induced revascularization of human umbilical vein endothelial cells (HUVEC) (Monteforte et al., 2016) and to act as a VEGF co-receptor in tumor angiogenesis (Aikawa et al., 2008; Whipple et al., 2012).

The aim of the present study was to identify growth factors secreted by KORS during HF angiogenesis and the involvement of GPC1.

## 2 Materials and Methods

### 2.1 Ethics Statement

Human scalp samples were obtained by Alphenyx (Marseille, France) by biopsy during human donor surgeries following informed consent. Applicable ethical guidelines and regulations were provided by Alphenyx.

### 2.2 Immunohistochemistry

The human HFs were isolated from human scalp according to Philpott’s method (Philpott et al., 1996). The HFs were embedded individually in Tissue-Tek OCT compound and quick frozen at −80°C. Longitudinal sections of the HFs were sliced with a cryostat. Sections were placed on glass slides and air-dried. Sections were then fixed in acetone for 10 min at −20°C. After several washes in PBS, the sections were placed in a serum solution. Primary antibody anti-GPC1 (Proteintech, Rosemont, IL, United States) was incubated overnight at 4°C. After several washes with PBS, the secondary antibody coupled with Alexa 488 was applied for 45 min at room temperature and in darkness. The Evans blue counterstain was applied after several washes for 5 min at room temperature. After the final washes, the glass slides were mounted under a coverslip using Fluoprep. The observations were realized using confocal microscope (TCS-SPE, Leica, Nanterre, France).

### 2.3 Cell Types

The cell types are described in Table 1. All cells were cultured at 37°C with 5% CO_2_ and used from passages 1 to 4 throughout the study.

**TABLE 1:** Cell types used.

Cell types	Suppliers (batch)	Culture medium
Human hair follicle dermal papilla cells (HHFDPC)	PromoCell (403Z014.6)	Mesenchymal stem cell medium (MSCM) +5% FBS and growth factors
Keratinocytes of outer root sheath (KORS)	ScienCell (9265)	Mesenchymal stem cell medium (MSCM) +5% FBS and growth factors
Human dermal microvascular endothelial cells (HDMEC)	ScienCell (2622)	Endothelial cell medium (EC_M_) +5% FBS and growth factors

### 2.4 Cell Starvation and Conditioned Media Collection

During starvation, after reaching 70% of confluence, the cells were incubated in their respective medium without FBS and growth factors. After 24 or 48 h, each type of conditioned medium was collected and stored at −80°C for further experiments.

### 2.5 Proliferation Assay

The WST-1 assay (Cell Proliferation Reagent WST-1, Roche, Basel, Switzerland) was performed to investigate cell proliferation. Human dermal microvascular endothelial cells (HDMECs) were seeded (2.5 × 103 cells/well) on 96-well plates and incubated for 24 h. Then, the medium of interest was added and incubated for 24 or 48 h according to the experiment. After the 24 or 48 h of incubation, the HDMECs were incubated with WST-1 reagent for 30 min. The colorimetric reaction was assessed using a microplate reader (Mithras LB 940, Berthold Technologies) at 450 nm.

### 2.6 Wound-Healing Assay

HDMECs were seeded (4.9 × 10^4^ cells/well) in Culture-Insert 2 Well in μ-Dish 35 mm (IBIDI, Martinsried, Germany). After 24 h, the inserts were removed, and the medium of interest was added for 24 h. The surface covered by HDMECs was observed at different times under a phase-contrast microscope (10x, EVOS™, Fisher Scientific, Illkirch, France). The uncovered surface was measured using the macro Wound-Healing Tool in ImageJ software (NIH, Bethesda, Maryland, United States).

### 2.7 Pseudotube Formation

HDMECs were seeded (2 × 104 cells/well) on 48-well plates coated with 100 µL of cold Matrigel^®^ (VWR, Radnor, PA, United States) in the medium of interest. Pseudotube formation was allowed to proceed for as long as 5 h and observed with a phase contrast microscope (4x, EVOSTM). Different parameters (such as number of nodes, meshes, junctions, segments and total lengths at 5 h) were measured using the macro Wound-Healing Tool in ImageJ software (ImageJ NIH).

To study the effect of VEGF and hepatocyte growth factor (HGF) on HDMEC pseudotube formation, the basal ECM (control) was supplemented with 200 ng/ml of VEGF, HGF or a combination of both.

### 2.8 Specific GPC1 Down-Regulation by GPC1 siRNA Transfection of HDMEC

siRNA specific to human glypican-1 (SMARTpool^®^ GPC1, L-004303-02-0005) and negative control siRNA (non-targeting pool, D-001810-10-05), were purchased from Dharmacon (Chicago, IL, United States). The siRNA targets different regions of the GPC1 mRNA: 1st siRNA target sequence (5′-ucg​gag​agc​ugu​aca​cgc​a-3′), 2nd siRNA target sequence (5′-agg​cgg​aga​ucu​cgg​gug​a-3′), 3rd siRNA target sequence (5′-aaa​uac​aac​aca​gac​gau​a-3′) and 4th siRNA target sequence (5′-ccg​cac​ugc​aga​cgg​gaa​u-3′). After reaching 60-80% of confluence, HDMEC were transfected with the siRNA pools (15 nM) using the PromoFectin-HUVEC reagent (PromoCell, Heidelberg, Germany) according to the manufacturer’s instructions. GPC1 mRNA and protein expression was assessed 29 h after siRNA transfection by RT-PCR. This time was chosen in order to allow pseudotube formation by transfected HDMEC. Indeed, 24 h after siRNA transfection, HDMEC were detached and seeded on Matrigel^®^ to form pseudotubes for 5 h. Two controls were performed as follows: HDMEC were incubated with the basal medium only (non-transfected) or with the negative control siRNA (siControl, siCTL).

To check whether the decrease of GPC1 expression was still significant in siRNA GPC1 transfected HDMEC after pseudotube formation, the HDMEC were collected and RNA was isolated to perform RT and qPCR to assess GPC1 gene expression (data not shown).

### 2.9 GPC1 Cleavage by Phospholipase C Treatment

In the wound-healing assays and pseudotube experiments, 0.5 unit per mL (U/mL) of PLC was added to basal ECM or the medium of interest, for 1 h at 37°C. For the pseudotube formation experiments, PLC preincubation was realized in cell suspension for 1 h at 37°C. Then, 0.1 U/mL of PLC was added until the end of the experiments.

### 2.10 Real-Time Reverse Transcription-Polymerase Chain Reaction

Total RNA was extracted using PureLinkTM RNA mini kit (Thermo Fisher Scientific, Waltham, MA, United States), and 250 ng of total RNA were retrotranscribed into cDNA using the Maxima first-strand cDNA synthesis kit with dsDNase (Thermo Fisher Scientific, Waltham, MA, United States). Real-time RT-PCR was performed using the Maxima SyBr green/ROX kit (Thermo Fisher Scientific, Waltham, MA, United States), and fluorescence detection was carried out with Agilent MX300P device and MxPro software (Santa Clara, CA, United States). The relative gene expression (normalized to housekeeping genes) was calculated by the ΔCt method. The Ct (threshold cycle) of the gene of interest was compared with average of the Ct of three different reference genes: peptidylprolyl isomerase A (PPIA), succinate dehydrogenase A (SDHA) and TATA binding protein (TBP), according to the method of Kozera and Rapacz (Kozera and Rapacz, 2013). Relative quantitative expression was determined as 2^–ΔCt^. The primers used in this study are presented in Table 2.

**TABLE 2:** Primers used for real-time RT-PCR.

Genes	Forward primers	Reverse primers
GPC1	5′-TGC​CCT​GAC​TAT​TGC​CGA​A-3′	5′-CAT​GGA​GTC​CAG​GAG​GTT​CCT-3′
GPC3	5′-GCC​CAT​TCT​CAA​CAA​CGC​CA-3′	5′-TGT​AGC​CAG​GCA​AAG​CAC​TA-3′
GPC4	5′-AGC​GGT​TGC​GGG​AGA​TGT​CGT-3′	5′-AGT​CAC​GAG​ACC​CCG​GCA​GTG-3′
GPC5	5′-GGC​ATG​GTT​GAA​CAA​GTC​AG-3′	5′-GCC​AGT​GTC​TGT​TTG​ATG​GA-3′
GPC6	5′-AGA​GCG​ACT​GGA​GGG​GCC​ATT-3′	5′-TTC​AGG​AGC​TGA​GCG​GGC​AGA-3′
PPIA	5′-GCA​GAC​AAG​GTC​CCA​AAG​AC-3′	5′-ACC​ACC​CTG​ACA​CAT​AAA​CC-3′
SDHA	5′-TGG​GAA​CAA​GAG​GGC​ATC​TG-3′	5′-CCA​CCA​CTG​CAT​CAA​ATT​CAT​G-3′
TBP	5′-TGC​ACA​GGA​GCC​AAG​AGT​GAA-3′	5′-CAC​ATC​ACA​GCT​CCC​CAC​CA-3′

The forward and reverse sequences of each primer are presented. The expression of the cerebroglycan, GPC2, was not studied because it is specifically expressed in neuronal differentiation (Stipp et al., 1994).

### 2.11 Immunoblotting

Cells were lysed in RIPA buffer (Sigma-Aldrich, Saint-Louis, MO, United States) supplemented with 1% protease inhibitor cocktail. Cell lysates were incubated 20 min on ice and mixed every 5 min; then, cell debris were precipitated by centrifugation at 10,000 g for 10 min at 4°C, and the supernatant of total cell protein extract was collected. To verify the cleavage of GPC1 by PLC, HDMECs were incubated with 0.5 unit per mL (U/mL) of PLC for 1 h at 37°C. Then, the medium was collected and the total proteins were isolated as previously described.

The samples were added to polyacrylamide gels as previously described (Perrot et al., 2019). The primary antibodies used in this study are presented in Table 3. The appropriate peroxidase-coupled secondary antibodies (1/10,000) were the anti-rabbit NA934V (GE Healthcare Life Sciences, Marlborough, MA, United States) and the anti-mouse NA931V (GE Healthcare Life Sciences).

**TABLE 3:** List of primary antibodies used in immunoblotting and co-immunoprecipitation experiments.

Recognized proteins	Host and isotype	Dilution or concentration	References
Actin	Polyclonal rabbit	1/2,000	A2066, Sigma-Aldrich
ADAM17	Polyclonal rabbit IgG	1 μg/ml	ab 2051, Abcam
c-Met	Monoclonal rabbit IgG	1/1,000	8198, Cell Signaling Technology
GPC1	Polyclonal rabbit IgG	1/1,000	16700-1-AP, Proteintech
HGF	Monoclonal rabbit IgG	1/1,000	52,445, Cell Signaling Technology
SDC1	Monoclonal mouse IgG2a	1/500	60185-2-Ig, Proteintech
VEGF	Polyclonal rabbit IgG	1/1,000	sc-507, Santa Cruz Biotechnology, Dallas, TX, United States
VEGFR2	Monoclonal rabbit IgG	1/1,000	9698, Cell Signaling Technology

### 2.12 Antibody Array

The growth factors in the conditioned media were determined by dot blotting (ab134002, Abcam, Cambridge, United Kingdom) according to the manufacturer’s instructions. Briefly, the membranes were saturated and incubated with 1 ml of the medium of interest for 2 h at room temperature. After three washes, the membranes were incubated with 1 ml biotin-conjugated anti-cytokines overnight at 4°C and were visualized by chemiluminescence (Detection Buffer C and D) using ChemiDoc MP (Bio-Rad, Hercules, CA, United States).

According to the manufacturer’s instructions, the receptors on the cell membranes were studied by dot blotting (ab193662 and ab134002, Abcam). After protein extraction of the cells of interest in the cell lysis buffer provided in the kit, the protein concentration was adjusted to 250 μg of protein in 1 ml of blocking buffer. Then, the same protocol was applied as described above.

### 2.13 Co-Immunoprecipitation

For co-immunoprecipitation experiments, protein lysates were incubated overnight at 4°C with Sepharose beads (protein A-Sepharose^®^ 4B, Sigma-Aldrich) and the anti-GPC1 antibody (16700-1-AP, Proteintech). First, the beads were saturated overnight in PBS supplemented with 1% BSA at 4°C. After three rinses with the Extraction Buffer, the beads were incubated overnight with 2 μg of the antibody of interest and 25 μg of samples at 4°C. The samples were analyzed by immunoblotting as described above. Two antibodies were used for the immunoblotting: anti-VEGFR2 (9698, Cell Signaling Technology, Danvers, MA, United States) and anti-c-Met (8198, Cell Signaling Technology) antibodies.

### 2.14 Statistical Analysis

Statistical analyses were performed using SatEL software (ad Science, Paris, France). Experiments were analyzed using Kruskal-Wallis test for unpaired nonparametric samples to compare all the groups. Then, Mann-Whitney *U* test was performed for a pairwise comparison. A *p* value less than 0.05 was considered significant. The respective *p* values are indicated in the figures as follows: *p < 0.05, **p < 0.01, ***p < 0.001, ****p < 0.0001, and *****p < 0.00001.

## 3 Results

### 3.1 GPC1 Is Expressed in a Specific Hair Follicle Area and in Endothelial Cells

**FIGURE 1:**
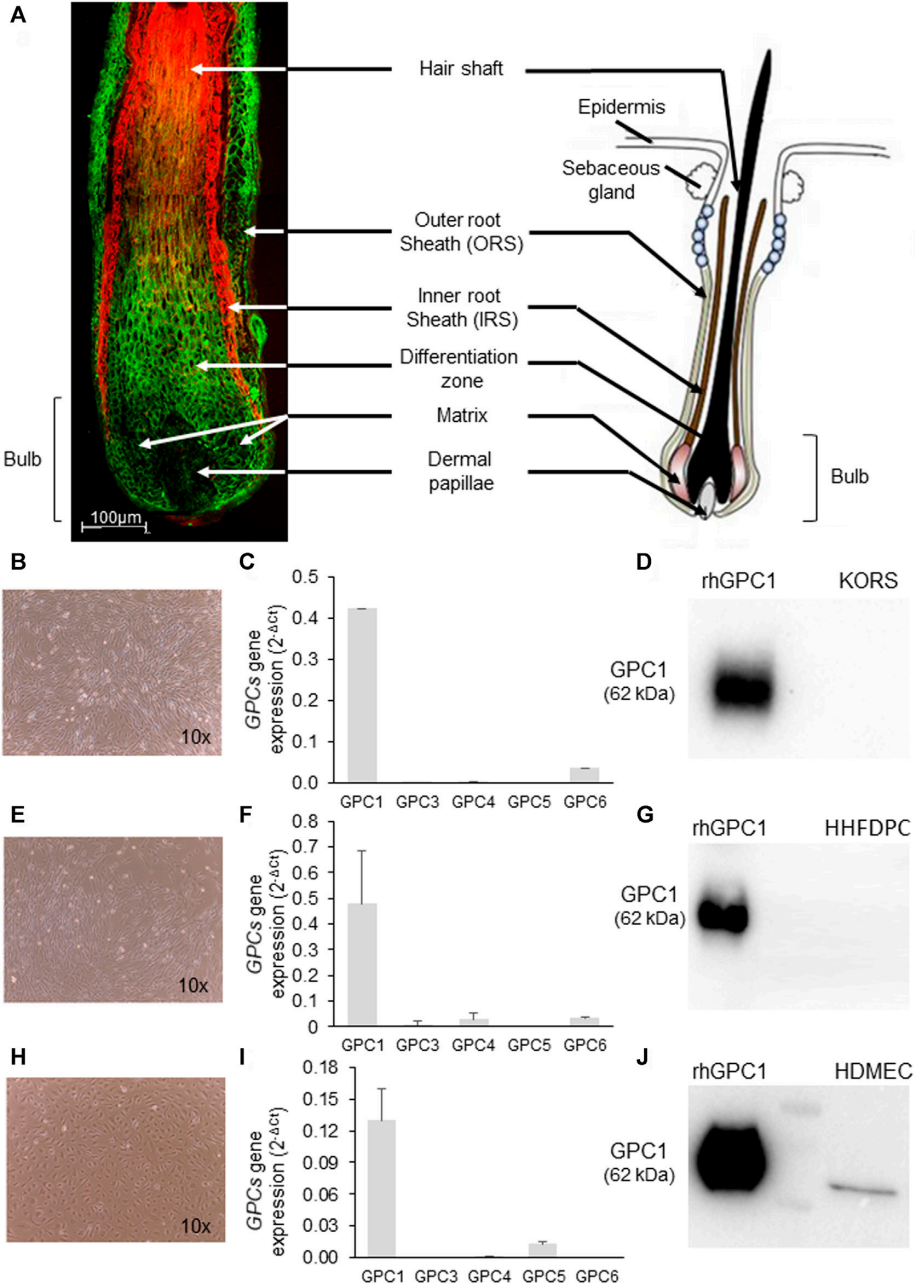
Characterization of GPC1 expression in hair follicle sections and in KORS, HHFDPC and HDMEC cultures. **(A)** Representative GPC1 labeling (green) in hair follicle sections counterstained with Evans blue dye (red) of a 64-year-old donor. A schematic representation of a hair follicle is presented (adapted from Sada and Tumbar, 2013 (1)). **(B–D)** Cell morphology (10x), GPC gene expression analyzed by real time RT-PCR (mean ± SEM, *n* = 10) and GPC1 protein expression analyzed by Western immunoblotting of KORS, respectively. **(E–G)** Cell morphology (10x), GPCs gene expression analyzed by real-time RT-PCR (mean ± SEM, *n* = 10) and GPC1 protein expression analyzed by Western immunoblotting of HHFDPCs, respectively. **(H–J)** Cell morphology (10x), GPCs gene expression analyzed by real time RT-PCR (mean ± SEM, *n* = 10) and GPC1 protein expression analyzed by Western immunoblotting of HDMECs, respectively.

Strong GPC1 labeling was observed in the outer root sheath and in the matrix of the HF but not in the inner root sheet. In dermal papilla, a very faint labeling of GPC1 protein was detected (Figure 1A). A conform cell morphology of KORS, human hair follicle dermal papilla cells (HHFDPCs), and HDMECs was observed *in vitro* (Figures 1B,E,H). A high level of *GPC1* gene expression was found in the KORS and HHFDPCs (Figures 1C,F). *GPC1* was the major glypican gene expressed in both cell types (Figures 1C,F). In contrast, the GPC1 protein was not detected by immunoblotting after 24 h of culture in complete medium (Figures 1D,G). In the HDMEC primary culture, *GPC1* was shown to be the most expressed glypican gene (Figure 1I), and GPC1 protein expression was detected (Figure 1J).

**FIGURE 2:**
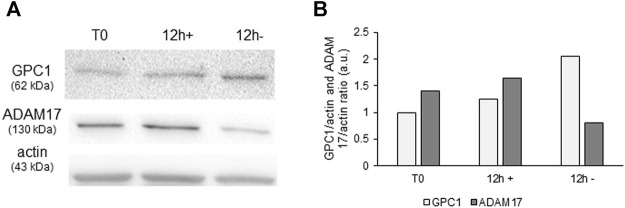
Modulation of ADAM17 protein expression and of GPC1 shedding in starvation condition. GPC1 and ADAM17 protein expression **(A)** and quantification **(B)** in KORS with (+) or without (−) serum (12 h) were analyzed by Western immunoblotting.

After 24 h of starvation, a high expression of GPC1 in both cell types (KORS and HHFDPC) was detected, while it was not detected in full medium condition (Supplementary Figure S1). Moreover, the expression of GPC1 and ADAM17 in KORS was analyzed after 12 h of starvation. It can be noticed that, after 12 h of starvation, GPC1 expression was inversely correlated with the detection of ADAM17 expression. Indeed, GPC1 expression increased in the KORS while ADAM17 expression was decreased (Figure 2).

### 3.2 Characterization of the Cell Communication

**FIGURE 3:**
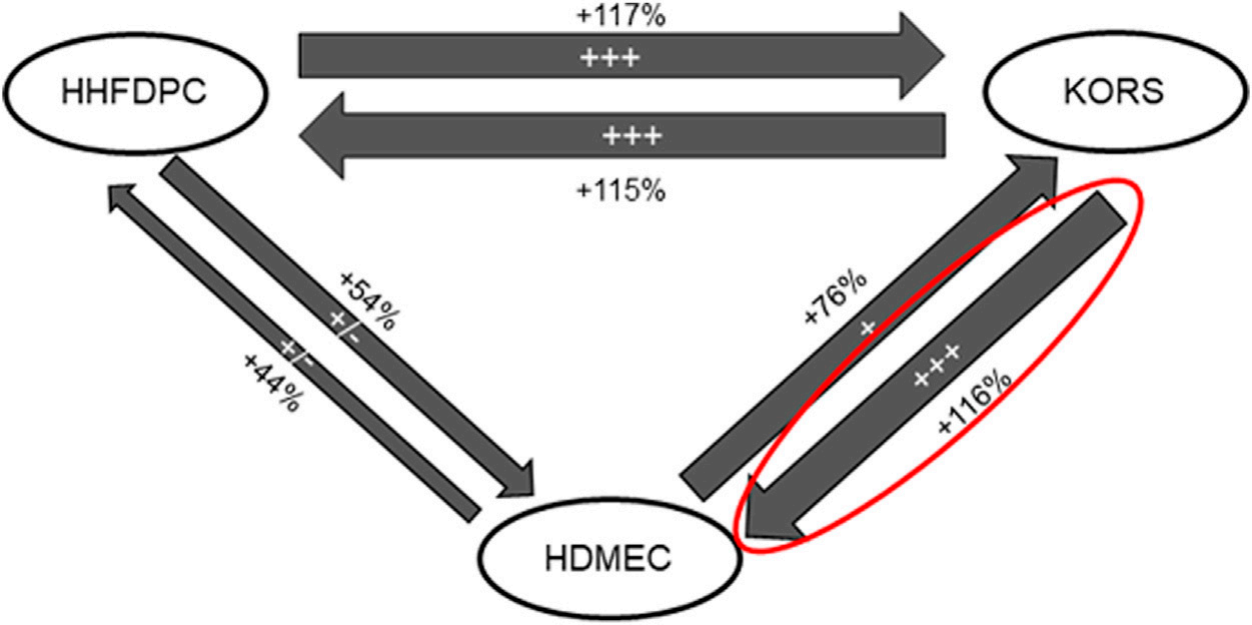
Diagram summarizing the effect of conditioned media from HHFDPC, KORS and HDMEC cultures on the proliferation of each cell type. The KORS_CM_ had the most significant effect on the HDMEC proliferation (*n* = 8 replicates and three independent experiments).

The study was devoted to characterizing the cell communications between KORS, HHFDPCs and HDMECs for a better understanding of the regulation of HF microvascular remodeling. The respective effects on cell proliferation between the 3 cell types are illustrated in Figure 3. Compared to the HHFDPC conditioned media (HHFDPC_CM_), the KORS conditioned media (KORS_CM_) greatly increased the HDMEC proliferation (54 and 116%, respectively). Thus, the effect of KORS_CM_ on HDMEC behaviors was further investigated. Nevertheless, a supplementary figure (Supplementary Figure S2) was added showing the graphs corresponding to the reciprocal effects of the 3 cell types in proliferation assays.

### 3.3 KORS-Conditioned Medium Stimulates the Proliferation, GPC1 Protein Expression, Migration, and Pseudotube Formation of HDMECs

**FIGURE 4:**
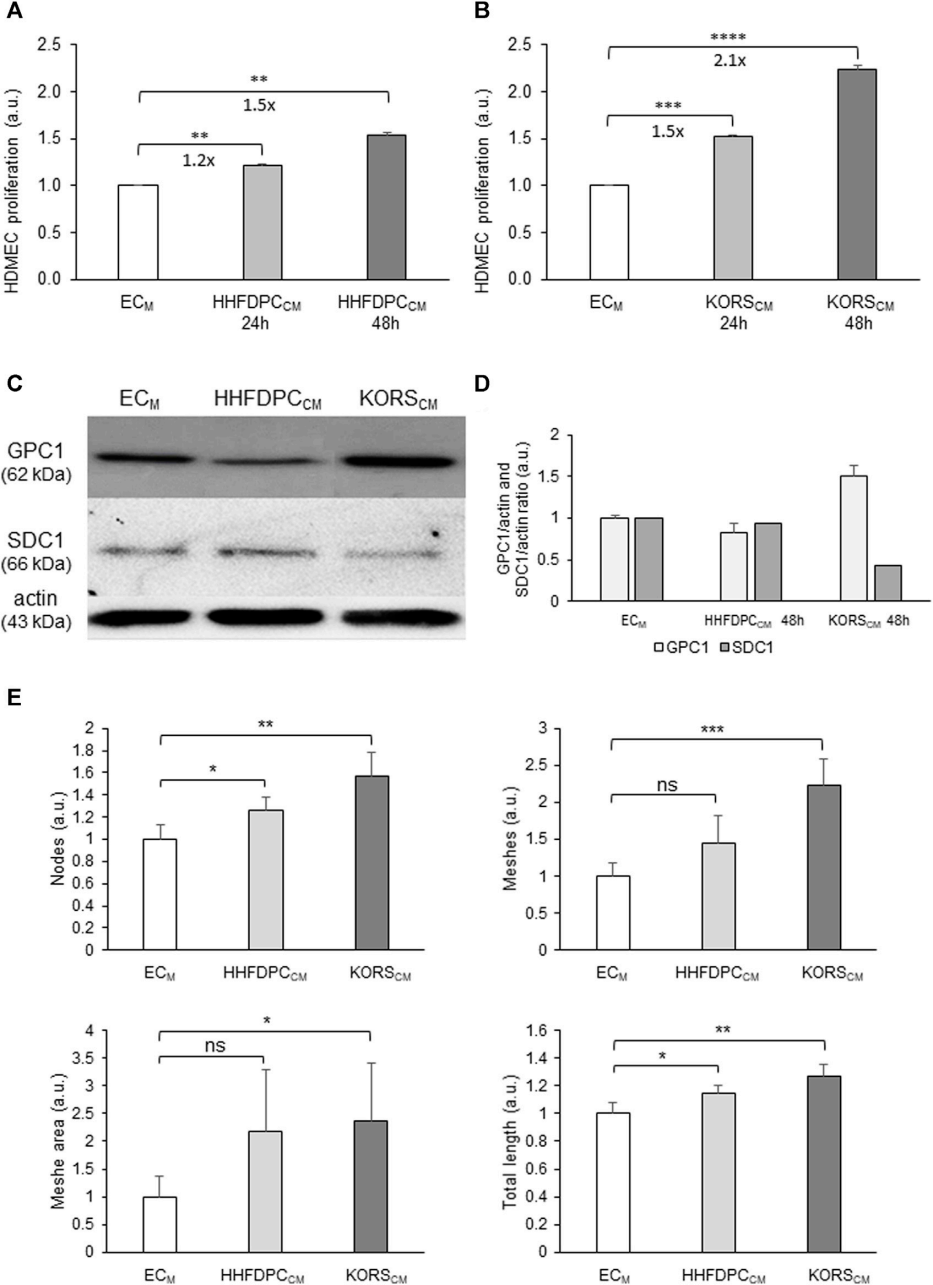
Effect of HHFDPC_CM_ and KORS_CM_ on HDMEC behaviors. **(A,B)** HDMEC proliferation was measured by colorimetric assay using WST-1 dye in presence of EC_M_ basal cell culture medium or HHFDPC_CM_ **(A)**, and in presence of EC_M_ or KORS_CM_ **(B)** for 24 or 48 h and expressed as the mean ± SEM, *n* = 8 replicates and three independent experiments **(C,D)**. Protein expression and quantification of GPC1 and SDC1 in the HDMECs as determined by Western immunoblotting. The cells were incubated in HHFDPC_CM_ or KORS_CM_ for 48 h before the analysis. The results are expressed as the mean ± SD, *n* = 2 for SDC1 and four independent experiments for GPC1. **(E)** Comparison of the effects of the HHFDPC_CM_ and KORS_CM_ (24 h of incubation) on HDMEC pseudotube formation. The KORS_CM_ had the most significant effect on HDMECs. Mean ± SD, *n* = 3 replicates and two independent experiments.

The effects of HHFDPC-conditioned media (HHFDPC_CM_) and KORS-conditioned media (KORS_CM_) on HDMEC proliferation, GPC1 protein expression and pseudotube formation were compared (Figure 4).

The HHFDPC_CM_ induced a significant increase in HDMEC proliferation, by 1.2-folds after 24 h and 1.5-folds after 48 h (Figure 4A). In the KORS_CM_, HDMEC proliferation was significantly increased, by 1.5-folds after 24 h and 2.1-folds after 48 h (Figure 4B).

The effects of HHFDPC_CM_ and KORS_CM_ on GPC1 protein expression in HDMECs were also compared to the control HDMECs in EC_M_. In contrast to the effect of HHFDPC_CM_, the KORS_CM_ increased the expression of GPC1 in HDMECs after 48 h (Figures 4C,D). In addition, syndecan-1 (SDC1) protein expression in the HDMECs was not increased in HHFDPC_CM_ or KORS_CM_ after 48 h (Figures 4C,D).

Both KORS_CM_ and HHFDPC_CM_ increased pseudotube formation as shown in Figure 4E.

KORS_CM_ exhibits stronger effect than HHFDPC_CM_ on HDMEC cells. Thus, the effect of KORS_CM_ only was investigated on HDMEC behavior using functional assays.

**FIGURE 5:**
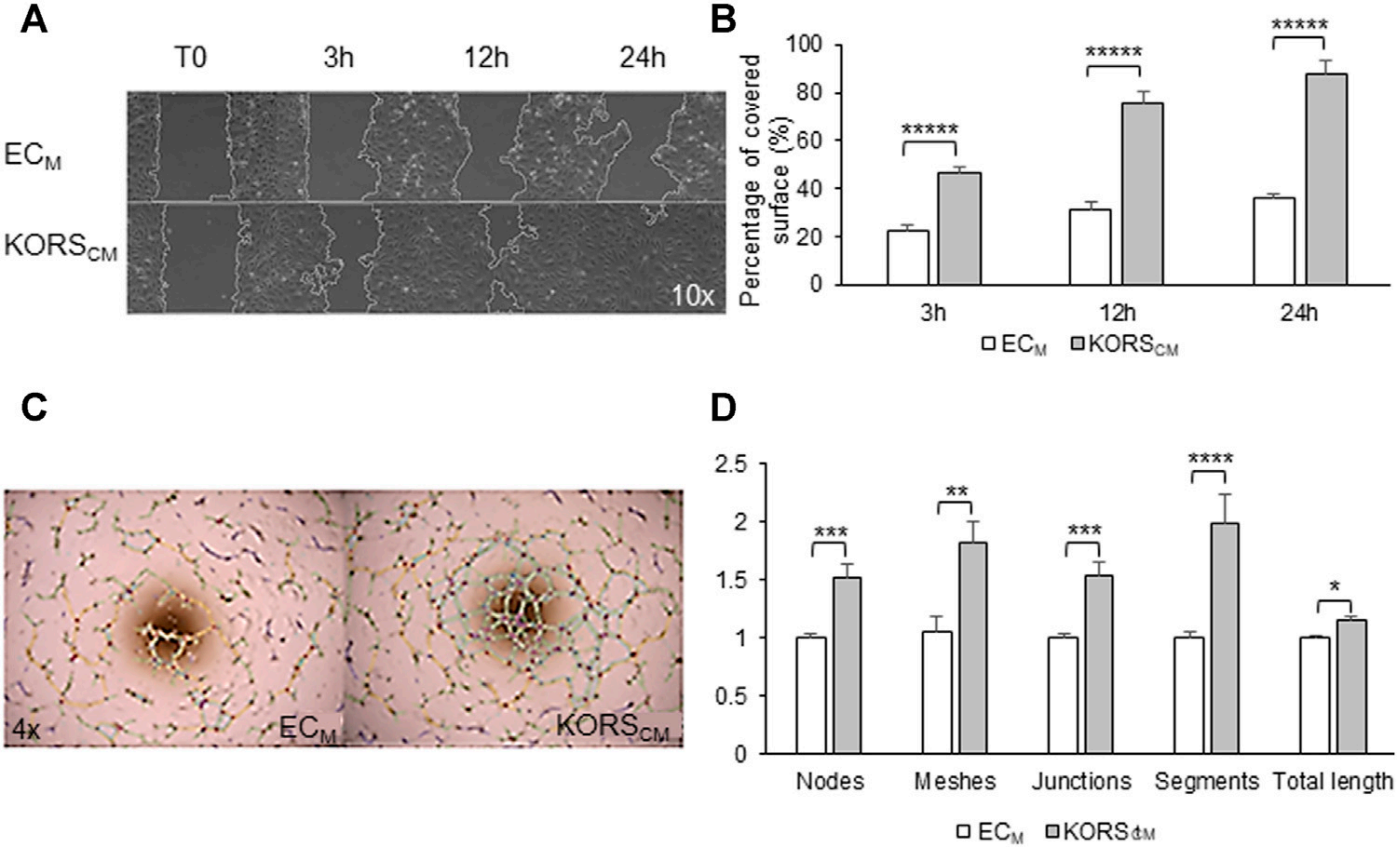
Angiogenic effect of KORS_CM_ on HDMEC. **(A,B)** The migration of HDMECs in the EC_M_ or KORS_CM_ is shown **(A)** and quantified as the percentage of recovery **(B)**. The results are expressed as the mean ± SEM, *n* = 3 replicates and three fields were analyzed per replicate, two independent experiments. **(C,D)** HDMEC pseudotube formation after 5 h in EC_M_ or KORS_CM_ is illustrated **(C)** and the number of nodes, meshes, junctions, segments, and the total length were calculated and expressed as the mean ± SEM, *n* = 8 replicates and three independent experiments **(D)**.

Wound-healing assays were performed (Figures 5A,B, Supplementary Movies S1, S2). In the control medium, HDMECs migrated and covered approximately 30% of the wound area after 24 h. In KORS_CM_, a strong and significant increase of HDMEC migration was observed. Indeed, a 50% coverage of the wound area (a twofold increase) was observed at 3 h. The kinetics of the wound healing showed that 80% (2.5-fold increase) and 90% (3-fold increase) of the wound area were covered by HDMECs at 12 and 24 h, respectively.

HDMEC pseudotube formation assays were also performed, and after 5 h of cell incubation, observations were made (Figures 5C,D). In this test, KORS_CM_ significantly increased the HDMEC ability to form pseudotubes. All analyzed parameters (number of nodes, meshes, junctions, segments, and total lengths) were significantly increased after 5 h of incubation in KORS_CM_.

### 3.4 GPC1 Is a Key Regulator of HDMEC Pseudotube Formation Induced by KORS-Conditioned Medium

**FIGURE 6:**
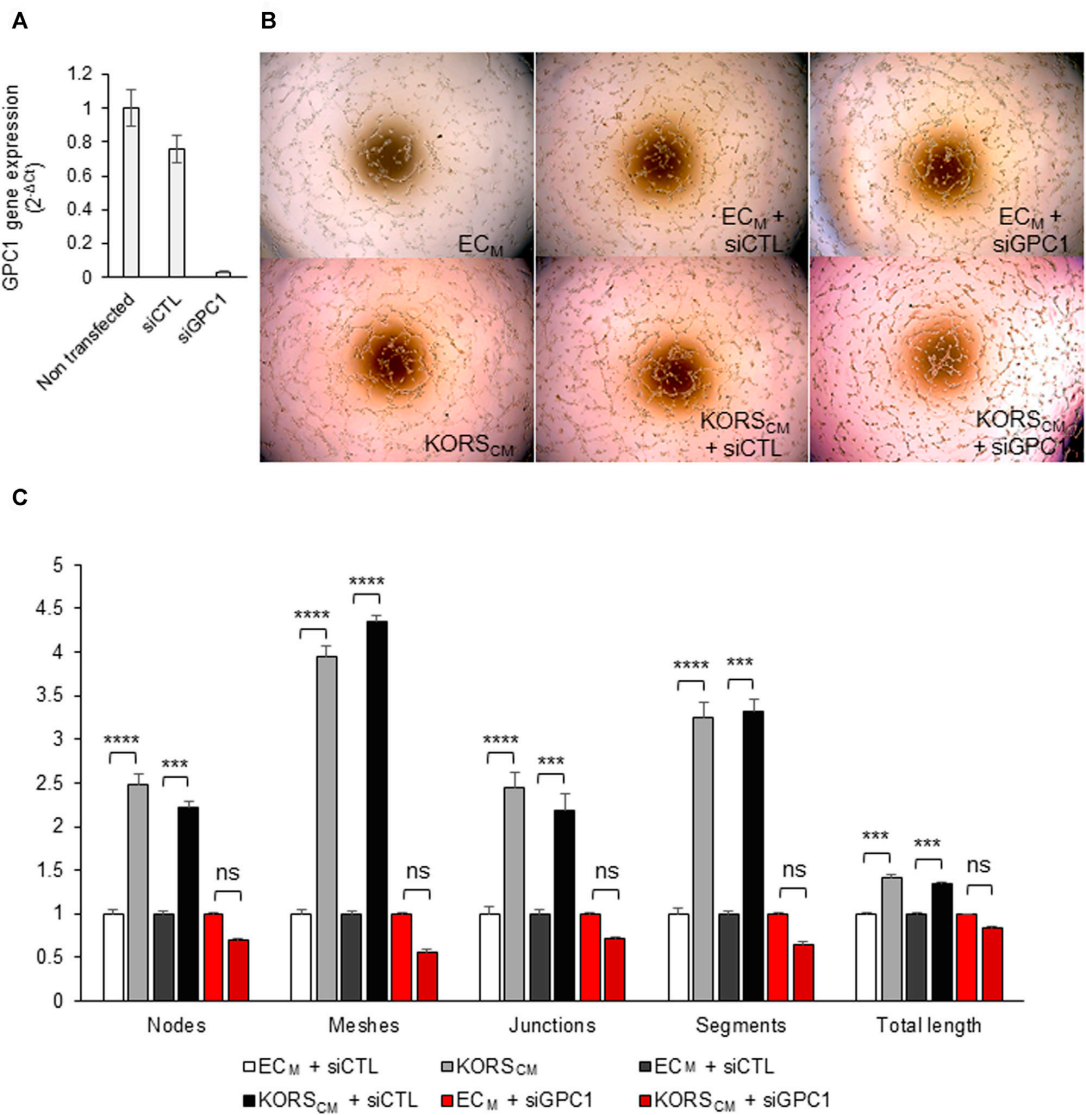
Down regulation of GPC1 gene expression by siRNA abolished the KORS_CM_-induced pseudotube formation by HDMEC. **(A)** GPC1 gene expression in HDMEC were analyzed by RT-qPCR. **(B,C)** Pseudotube formation of non-transfected or transfected (siCTL or siGPC1) HDMECs after 5 h in EC_M_ or KORS_CM_ is illustrated **(B)** and the number of nodes, meshes, junctions, segments, and the total length were calculated and expressed as the mean ± SEM, *n* = 4 to 8 replicates and three independent experiments **(C)**.

To study the role of GPC1 in HDMEC pseudotube formation induced by KORS_CM_, HDMEC were transfected with GPC1 siRNA (siGPC1) (Figure 6). A strong decrease of GPC1 gene expression as compared to non-transfected cells (−96.7%) or to siControl (siCTL) (−73.4%) was observed 29 h after GPC1 siRNA transfection (Figure 6A).

Non-transfected and siRNA-transfected HDMECs were tested in pseudotube formation assays. The induction of pseudotube formation by the KORS_CM_ was totally abrogated by the drastic down-regulation of gene expression of GPC1 by GPC1 siRNA (Figures 6B,C).

### 3.5 Anchored GPC1 Promotes HDMEC Migration and Pseudotube Formation Induced by KORS-Conditioned Medium, While Cleaved GPC1 Promotes HDMEC Proliferation

**FIGURE 7:**
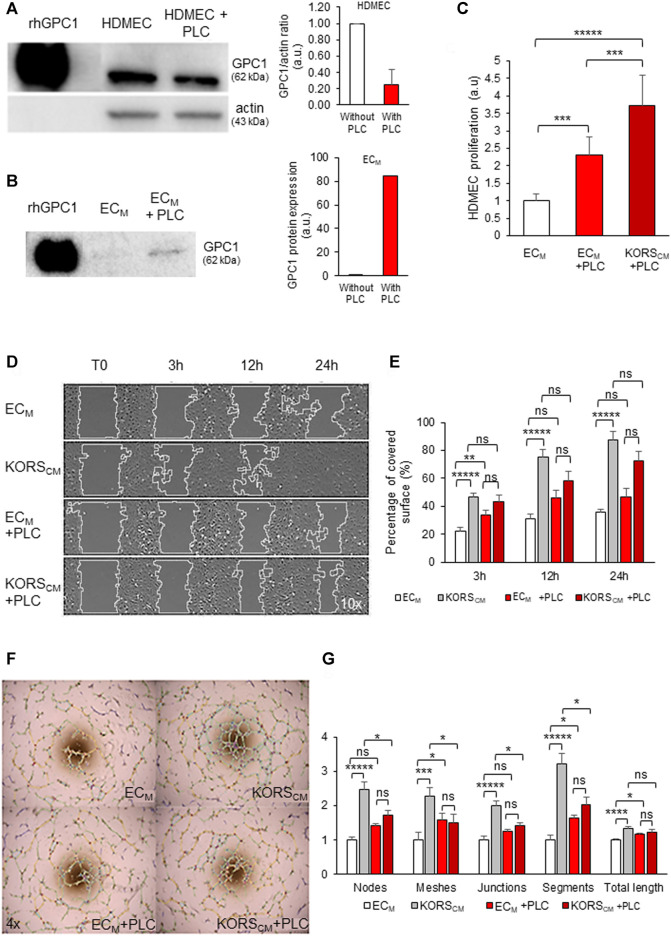
Effect of PLC incubation on HDMEC proliferation, migration and pseudotube formation. **(A,B)** HDMECs were treated with 0.5 U/mL PLC for 1 h at 37°C. Total cell proteins **(A)** and conditioned EC_M_ medium proteins **(B)** were extracted, analyzed by Western immunoblotting and quantified for GPC1 protein expression. **(C)** HDMEC proliferation was measured by colorimetric assay using WST-1 dye in basal EC_M_, EC_M_ + PLC or KORS_CM_ + PLC for 24 h and expressed as the mean ± SD, *n* = 8 replicates and two independent experiments. **(D,E)** The migration of HDMECs in EC_M_ and KORS_CM_ with or without PLC (24 h of incubation) was observed **(D)** and quantified as percentage of recovery **(E)**. The results are expressed as the mean ± SEM, *n* = 3 replicates and three fields were analyzed per replicate, two independent experiments **(E)**. **(F,G)** Pseudotube formation of HDMECs in EC_M_ and KORS_CM_ with or without PLC as observed 5 h after seeding **(F)**. The number of nodes, meshes, junctions, segments, and the total length were quantified and expressed as the mean ± SEM, *n* = 5 replicates and three independent experiments **(G)**.

The glypicans are known to regulate growth factors both under anchored and cleaved forms. PLC was used to cleave the GPI anchor and to release GPC into the culture medium. GPC1 protein expression was measured by immunoblotting the HDMEC membrane protein extract and normalized to actin. The preincubation of HDMECs with PLC led to a 3-fold decrease in GPC1 expression in the membrane protein extract (Figure 7A). Concomitantly, the analysis of the EC_M_ showed that the GPC1 protein could not be detected under control conditions; it was detected only after PLC treatment (Figure 7B).

The HDMEC proliferation analysis showed that the cleavage of GPC1 by PLC treatment (Figure 7C) stimulated a 2-fold increase in cell proliferation in EC_M_ and a 4-fold proliferation increase when HDMECs were grown in KORS_CM_.

Incubation in KORS_CM_ significantly increased the migration of HDMECs (Figures 7D,E, Supplementary Movies S1–S4). However, there were no significant migratory differences in the HDMECs cultured in EC_M_ with PLC and KORS_CM_ with PLC.

When the HDMECs were incubated in KORS_CM_, the pseudotube formation was significantly increased (Figure 7F), as measured for all parameters shown in Figure 7G. After treatment with PLC, no significant difference was observed between the EC_M_ and KORS_CM_. Furthermore, the addition of PLC to the KORS_CM_ significantly decreased the pseudotube formation by HDMECs compared to pseudotube formation made by HDMECs incubated in the KORS_CM_ without PLC (Figures 7F,G).

### 3.6 KORS Regulate HDMEC Pseudotube Formation *via* HGF/C-Met and VEGF/VEGFR2 in Tripartite Complexes Associated to GPC1

**FIGURE 8:**
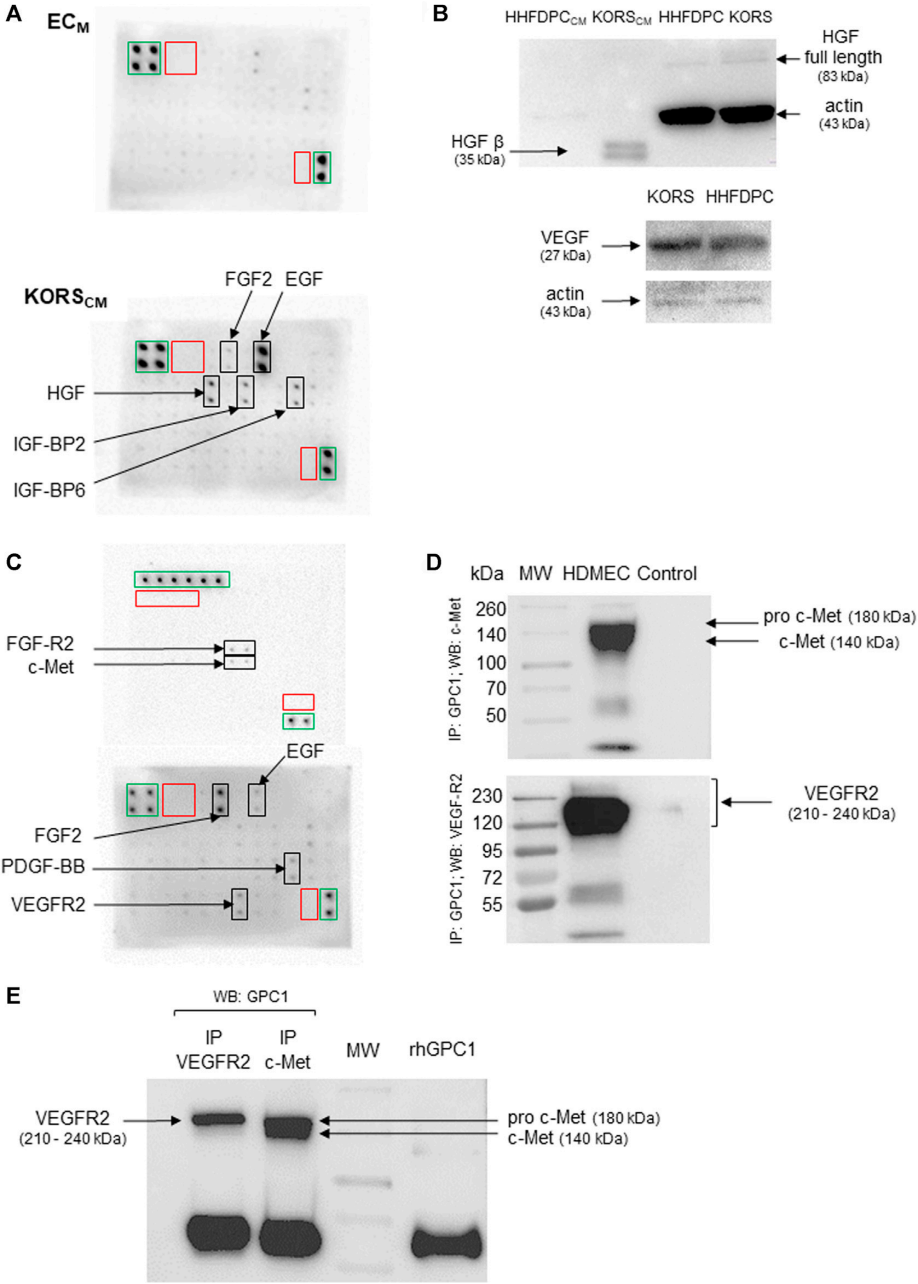
HDMEC express the receptors for VEGF and HGF, which are secreted by KORS. **(A)** Growth factors secreted by KORS analyzed by protein array from their conditioned medium (KORS_CM_) and compared to the basal cell culture medium (EC_M_) without cells. Green frames: positive control spots; red frames: negative control spots; black frames: spots corresponding to the indicated target. **(B)** The production of HGF and VEGF in the KORS were compared to that of the HHFDPCs by Western immunoblotting. **(C)** Growth factors and receptors of HDMECs analyzed by protein array. Green frames: positive control spots; red frames: negative control spots; black frames: spots corresponding to the indicated target. **(D)** The direct interaction between GPC1 with VEGFR2 or c-Met was analyzed by co-immunoprecipitation. A whole cell protein extract (25 μg) from the HDMECs was precipitated using anti-GPC1 antibody. Anti-c-Met or anti-VEGFR2 antibody was used to reveal the membrane. A control experiment without antibody was performed. **(E)** A reverse immunoprecipitation assay, corresponding to the co-immunoprecipitation shown in Figure 8D, was conducted from HDMEC whole cell protein extracts using anti-VEGFR2 or c-Met antibody. Then, the isolated immunocomplexes were immunoblotted using anti-GPC1 antibody.

Protein array analysis revealed traces of EGF and IGF-BP6 in the control EC_M_. EGF, HGF, IGF-BP2, IGF-BP6, and FGF2 were detected in the dot blot of the KORS_CM_ (Figure 8A). The protein analysis then focused on HGF, highlighted by dot blot assays and VEGF, known to regulate angiogenesis. In the HHFDPC and KORS extracts, HGF (full length) and VEGF were detected (Figure 8B). In contrast to the HHFDPC_CM_, the active form of HGF, HGF β, was specifically detected by immunoblotting in the KORS_CM_. Protein array analysis of HDMEC extracts revealed the presence of VEGFR2, c-Met, and FGFR2 receptors and the presence of FGF2 and PDGF-BB GFs (Figure 8C).

To verify whether GPC1 was involved in the proangiogenic effect of the KORS_CM_ on HDMECs, the direct interaction of GPC1 with c-Met and VEGFR2 was investigated in HDMEC by co-immunoprecipitation. After GPC1 precipitation, c-Met, the c-MET precursor form (pro-c-Met) and VEGFR2 were detected by immunoblotting (Figure 8D). Moreover, conversely, after precipitation with c-Met or VEGFR2 antibody, GPC1 was detected by immunoblotting (Figure 8E).

**FIGURE 9:**
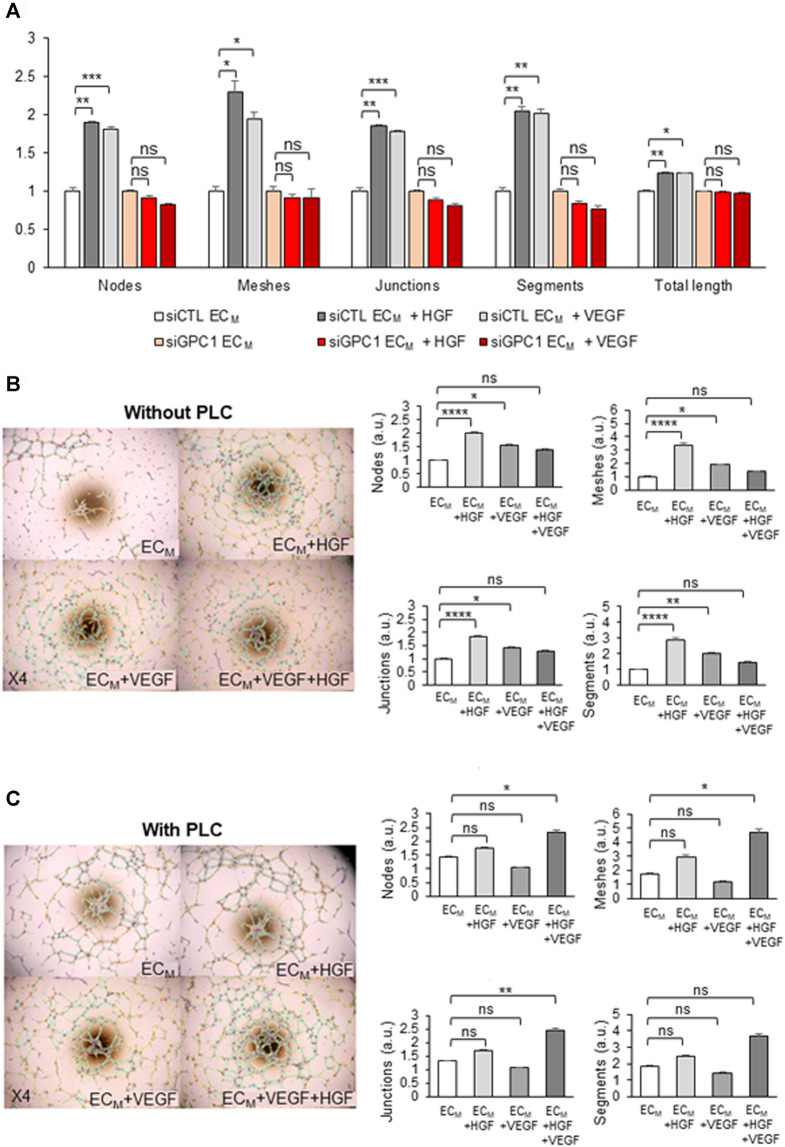
KORS regulate HDMEC pseudotube formation in a GPC1-dependent manner *via* HGF/c-Met and VEGF/VEGFR2. **(A)** Pseudotube formation of non-transfected or transfected (siCLT or siGPC1) HDMEC after 5 h in EC_M_ without or with VEGF or HGF were performed and the number of nodes, meshes, junctions, segments, and the total length were calculated and expressed as the mean ± SEM, *n* = 4 replicates and two independent experiments. **(B,C)** Effect of HGF, VEGF and the combination of both growth factors on HDMEC pseudotube formation without **(B)** or with **(C)** 0.5 U/mL PLC treatment for 1 h at 37°C. The number of nodes, meshes, junctions and segments was quantified. The results are expressed as the mean ± SEM in the right panel, *n* = 6 replicates and two independent experiments.

To study the role of GPC1 in HDMEC pseudotube formation induced by VEGF or HGF, non-transfected and siRNA-transfected HDMECs were tested in pseudotube formation assays. The induction of pseudotube formation by VEGF or HGF was totally abrogated by the drastic down-regulation of gene expression of GPC1 by GPC1 siRNA in HDMECs (Figure 9A).

Moreover, the effect of VEGF and/or HGF on HDMEC pseudotube formation was analyzed under two conditions: when GPC1 was anchored or cleaved. After analysis of the number of nodes, meshes, junctions, and segments of the pseudotube network (Figure 9B), HGF treatment was demonstrated to induce a significant increase of HDMEC pseudotubes formation. VEGF treatment was confirmed to increase significantly HDMEC pseudotube formation. Moreover, the effect of HGF on HDMEC pseudotube formation was shown to be more efficient than VEGF effect. However, the addition of HGF with VEGF in the EC_M_ did not induce either a cumulative or a synergistic effect on HDMEC pseudotube formation.

After PLC treatment, no significant differences in HDMEC pseudotube formation were observed between the control EC_M_ without supplements and with supplemented HGF or VEGF (Figure 9C). In contrast, in the EC_M_ supplemented with HGF and VEGF together, the HDMEC pseudotube formation was significantly increased in a synergistic manner.

## 4 Discussion

**FIGURE 10:**
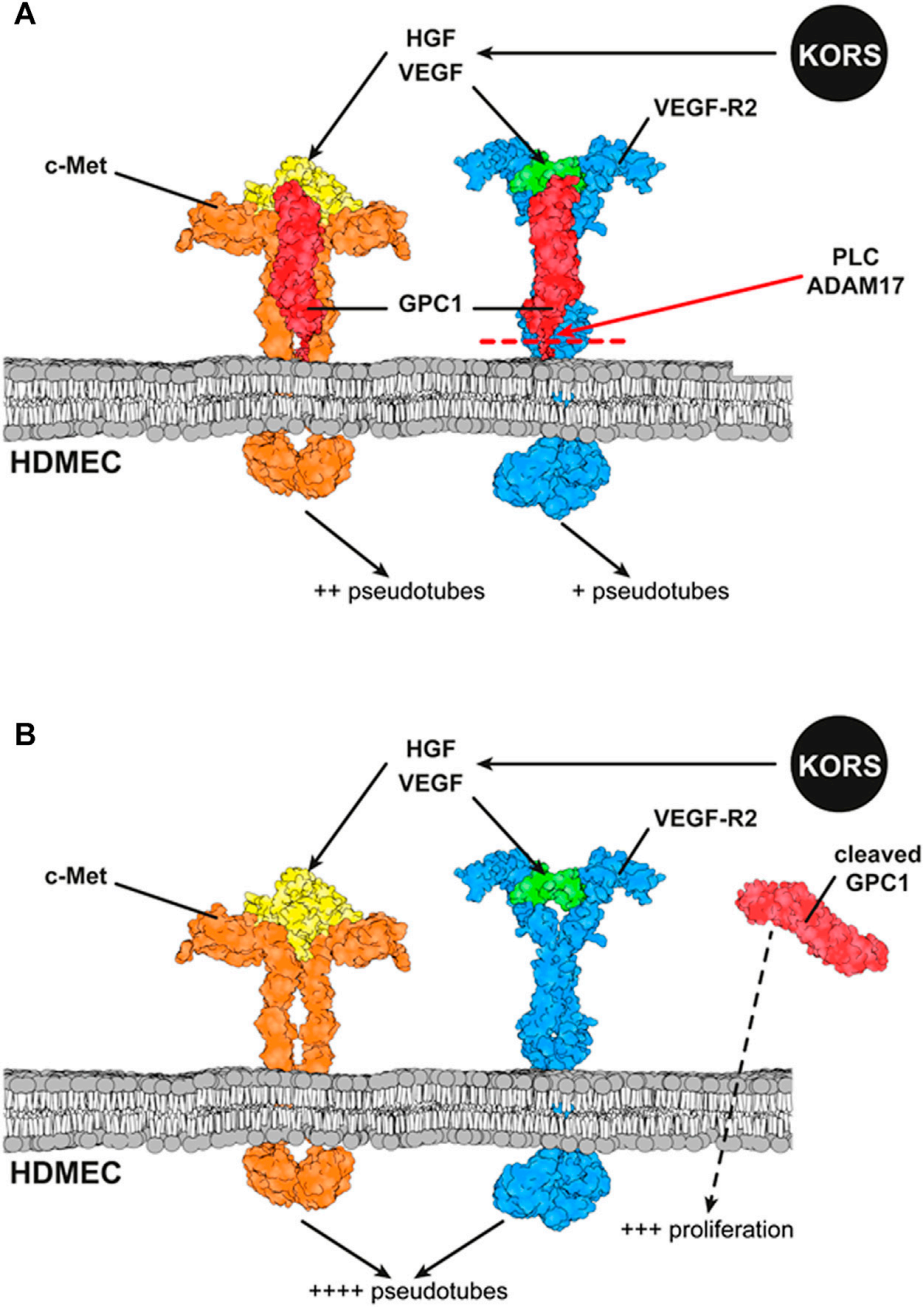
Schematic representation of interactions between GPC1, c-Met and VEGFR2 and their ligands at the HDMEC membrane. **(A)** The downstream effect of the binding of the ligands secreted by KORS to their specific receptors expressed on HDMECs and the interaction between the receptors are illustrated. The effects of VEGF and HGF on HDMEC pseudotube formation are presented. **(B)** Effects of GPC1 cleavage by PLC on HDMEC pseudotube formation and proliferation are illustrated.

Several studies have been conducted on the expression and distribution of HSPGs according to the phases of the HF cycle (Bayer-Garner et al., 2002; Malgouries et al., 2008; Wadstein et al., 2020). Moreover, to our knowledge, no analysis has been performed on the expression and function of GPCs in the different HF compartments. The present report shows for the first time, the expression of GPC in HFs. Among the six glypican members, *GPC1* is the major expressed glypican in HFs, with specific regulation of its expression in KORS, HHFDPCs, and microvascular endothelial cells. In case of alopecia, the microvascularization of the HFs decreases, favoring hair miniaturization. Several proteoglycans (Ozerdem and Stallcup, 2004; Albig et al., 2007; Niewiarowska et al., 2011) and glycosaminoglycans (Jung et al., 2001; van Wijk and van Kuppevelt, 2014) have been shown to regulate angiogenesis. This study suggests that GPC1 is involved in the HDMEC responses to growth factors secreted by KORS. GPC1, through formation of ternary complexes with VEGF/VEGFR2 and HGF/c-Met complexes, might modulate HDMEC proliferation or pseudotube formation. Altogether, these results are summarized in Figure 10.

The effect of KORS_CM_ in HDMEC behaviors was studied because the communication study demonstrates it has stronger effect than HHFDPC_CM_.

In isolated HF sections, GPC1 labeling was very faint in the dermal papilla, which is in agreement with the GPC1 protein being undetected by immunoblotting in the HHFDPC culture. However, the strong GPC1 protein labeling observed in the outer root sheath was not confirmed by immunoblotting in the KORS culture after 24 h in complete medium. Malgouries and his collaborators have shown that HSPG expression differs according to the phases of hair cycle (Malgouries et al., 2008). GPC1 protein expression can be detected in KORS after starvation. This result suggests that GPC1 protein expression is dependent on cell culture conditions and on starvation. This finding was previously described for other matrix proteins, including elastin, fibronectin or type I collagen (Wanjare et al., 2013). Moreover, the regulation of protein distribution at the cell surface may occur upstream, during mRNA translation (Cenik et al., 2015; Lu and Rothenberg, 2018), intracellular storage, and protein trafficking (Yamamoto et al., 2017), or downstream, during protein cleavage (Kawahara et al., 2017) or degradation (Hershko and Ciechanover, 1998). For example, *GPC1* mRNA translation is inhibited by the microRNA miR-96-5p (Li et al., 2014). In mouse, the miR-324-5p targets GPC1 to regulate Hedgehog (Hh) pathway (Woods et al., 2019). Chamorro-Jorganes and collaborators have demonstrated that the miR-149 regulates the angiogenic response to FGF2 witch is mediated by GPC1 (Chamorro-Jorganes et al., 2014). The hypermethylation of miR-149 modulates the GPC1 gene expression (He et al., 2014). GPC1 is a GPI-anchored proteoglycan which can be cleaved and released (Filmus et al., 2008). Several proteases cleave GPC1, including ADAM17 (Kawahara et al., 2017). KORS express ADAM17 protein, but this expression decreases under serum-free condition, in contrast to the GPC1 expression, which is increased. ADAM17 expression was already shown to depend on cell culture conditions, as demonstrated in chondrocytes (Flannery et al., 1999). The difference of ADAM17 expression in the present report may explain the difference of GPC1 detection by Western immunoblotting. In KORS, ADAM17 probably regulates GPC1 membrane protein shedding.

The regulation by growth factors of the major pathways of tissue or organ development and regeneration is impaired in the absence of glypicans (Kreuger et al., 2004; Ayers et al., 2010; Dwivedi et al., 2013; Yamamoto et al., 2013; Capurro et al., 2017). During embryonic development, glypicans have specific expression profiles and roles (Saad et al., 2017). GPCs regulate cell proliferation and numerous signaling pathways under physiological conditions (Saunders et al., 1997; Theocharis et al., 2010; Filmus and Capurro, 2014; Shi et al., 2020) and in cancer (Kleeff et al., 1998; Yiu et al., 2011; Gao et al., 2015a; Gao et al., 2015b; Cao et al., 2018; Theocharis and Karamanos, 2019). GPC1 regulates embryonic and cancer development (Qiao et al., 2003; Aikawa et al., 2008; Whipple et al., 2012; Chamorro-Jorganes et al., 2014; Li et al., 2014). It has been reported to interact with different growth factors, such as FGF2 and VEGF, and to play a role in angiogenesis (Monteforte et al., 2016). In the case of hair follicle vascularization, the role of GPC1 remained to be discovered. In this study, we have shown that GPC1 siRNA transfection abolished the effect of the KORS_CM_ on HDMEC pseudotube formation. This result demonstrated the specific key role of GPC1 for the regulation of hair follicle vascularization mediated by KORS. To go further, we wanted to analyze the effect of GPC1 released. After cleavage by PLC (Hereld et al., 1986), the role of GPC1 on HDMEC angiogenic potential was analyzed. GPC1 was shown to be the major GPCs expressed by HDMEC, suggesting that the effect observed after PLC treatment is mainly the fact of GPC1 shedding. However, we cannot exclude a cleavage of other GPCs or of other GPI-anchored molecules. The experiment using siRNA against GPC1 supports our hypothesis that the GPC1 cleavage by the PLC leads to the observed results on proliferation, migration and pseudotube formation. Our results showed that GPC1 is involved both in the proliferation and in migration/pseudotube formation of human endothelial cells. GPC1 shedding by PLC observed in the basal endothelial cell medium (EC_M_) which is very poor of growth factors can be more attributed to the direct effect of GPC1 loss rather than its capacity to sequester growth factors. In contrast, in KORS_CM_, we identified several growth factors which could be sequestered by the heparan sulfate glycosaminoglycan chains of GPC1. Indeed, the cleavage of GPC1 by PLC treatment increased HDMEC proliferation, abolished the effect of the KORS_CM_ on the migration of the HDMECs and inhibited the capability of HDMECs to form pseudotubes in KORS_CM_. Several studies have shown the implication of ADAM17 in the angiogenic processes (Rego et al., 2014; Caolo et al., 2015). For example, in three-dimensional collagen matrices, Kwak and collaborators showed that ADAM17 and TIMP-3 modulate endothelial invasion responses (Kwak et al., 2009). Moreover, ADAM17 is known to cleave GPC1 when it is induced by EGF (Kawahara et al., 2017). However, we have shown that KORS secrete EGF in the protein array analysis. Thus, further analyses on ADAM17 and EGF expression and function on HDMEC would be necessary to better understand the role of GPC1 shedding in the regulation of HDMEC angiogenesis.

In addition, the expression of GPC1 and SDC1, the latter being a full transmembrane HSPG, was compared in presence of KORS_CM_. The KORS_CM_ induced specifically GPC1 expression in HDMECs, as this medium did not alter the expression of SDC1. This result suggested that the major effect of KORS_CM_ was on the GPC1 expression and not on SDC1. Thus, KORS_CM_ is mainly and specifically on GPCs rather than SDCs both members of the family of membrane HSPGs. Similar to its role in glioma angiogenesis (Qiao et al., 2003), GPC1 appears to be a specific HSPG actor in hair microvascular remodeling induced by growth factors. Thus, KORS could increase the HDMEC response to growth factors by increasing GPC1 expression in HDMECs.

Previous studies have focused on VEGF secretion by the different HF compartments (Stenn et al., 1988; Lachgar et al., 1996; Kozlowska et al., 1998; Bassino et al., 2015; Idali, 2016). Moreover, Yano and collaborators showed a spatiotemporal correlation between VEGF expression by mouse keratinocytes and perifollicular angiogenesis, demonstrating the effect of KORS on the remodeling of the vascularization in mouse skin (Yano et al., 2001). In this report, the growth factors secreted by KORS with an angiogenic effect were identified. KORS are reported to express HGF (Shimaoka et al., 1995) and VEGF (Yano et al., 2001). For the first time, our results have demonstrated, that the active HGF β subunit (35 kDa) was highly and specifically secreted by KORS. Our results have shown that HGF has a stronger pro-angiogenic effect than VEGF on HDMECs. No synergistic or cumulative effect between these two growth factors was observed in the HDMECs, in contrast to the results described for HUVECs (Xin et al., 2001). This difference in findings may be related to different cell types and vasculature. HDMECs were used in our study because they exhibit cell morphology, phenotypes, and properties similar to those of endothelial cells in the hair microvasculature (Sakita et al., 1994). Regulation of the hair microvascularization remodeling by KORS is very similar to that observed in angiogenesis induced by mesenchymal stem cells (Kachgal and Putnam, 2011). Similarly, the activation of dental pulp stem cells by FGF2 induced the secretion of VEGF and HGF promoting angiogenesis (Gorin et al., 2016).

HSPG are known to form ternary complexes with growth factors and their receptors (Sasisekharan, 2000; Qiao et al., 2003; Rapraeger, 2013) and to facilitate the c-Met dimerization and activation (Zioncheck et al., 1995). For example, GPC1 specifically forms a ternary complex with FGF2 and FGF-receptor 1 to promote cell signaling pathway activation in glioma vessel endothelial cells (Qiao et al., 2003; Su et al., 2006). Moreover, GPC1 is known to promote VEGF-induced revascularization in HUVECs (Monteforte et al., 2016) and to act as a VEGF co-receptor in these cells (Aikawa et al., 2008). It is known that the angiogenic effect of VEGF differs according to the VEGF receptors. VEGFR1 is pro-proliferative, and VEGFR2 promotes cell migration (Simons et al., 2016). In the present study, GPC1 has been demonstrated to be a co-receptor for VEGF and HGF. It might be involved in the angiogenic potential of VEGF and HGF in HDMECs. The mandatory role of GPC1 for the induction of HDMEC pseudotube formation by VEGF or HGF was demonstrated by GPC1 siRNA transfection. GPC1 appears to form a ternary complex with VEGF and VEGFR2 or with HGF and c-Met to promote angiogenesis. Moreover, we compared the effect of anchored and cleaved GPC1. The cleavage of GPC1 strongly inhibited the pseudotube formation induced by HGF and totally abolished the pseudotube formation induced by VEGF. VEGFR2 and c-Met have a synergistic effect on pseudotube formation in this case. It is possible that cleaved GPC1 leads to an accumulation of HGF and VEGF close to their receptors and enhances their action (Ayers et al., 2010). The cleavage of GPC1 at the membrane may also lead to VEGFR2 and c-Met dimerization (Heldin, 1995; Wicker and Guillermo Suarez, 1996).

In conclusion, this study identifies, for the first time, that active HGF is secreted by KORS and regulates HF angiogenesis in association with GPC1. Depending on whether it is the anchored or cleaved form, GPC1 might promote the proliferation or migration of endothelial cells or the formation of pseudotubes. GPC1 acts as a co-receptor for VEGFR2 and c-Met and it is necessary for the induction of HDMEC pseudotube formation induced by KORS_CM_. Further work is needed to clarify the role of GPC1 glycosaminoglycan chains and the degree and type of their sulfation in the HF vascularization. Thus, GPC1 might constitute an interesting target to tackle alopecia in cosmetology research.

## Data Availability

The original contributions presented in the study are included in the article/Supplementary Material, further inquiries can be directed to the corresponding author.
