# Supplementary material for: The Glypican-1/HGF/C-Met and Glypican-1/VEGF/VEGFR2 Ternary Complexes Regulate Hair Follicle Angiogenesis
Source: Front Cell Dev Biol. 2021 Dec 8;9:781172. doi: 10.3389/fcell.2021.781172 (PMC8692797; doi:10.3389/fcell.2021.781172)
Supplement: Supplementary file 1 [file Presentation1.PPTX]

## Slide 1
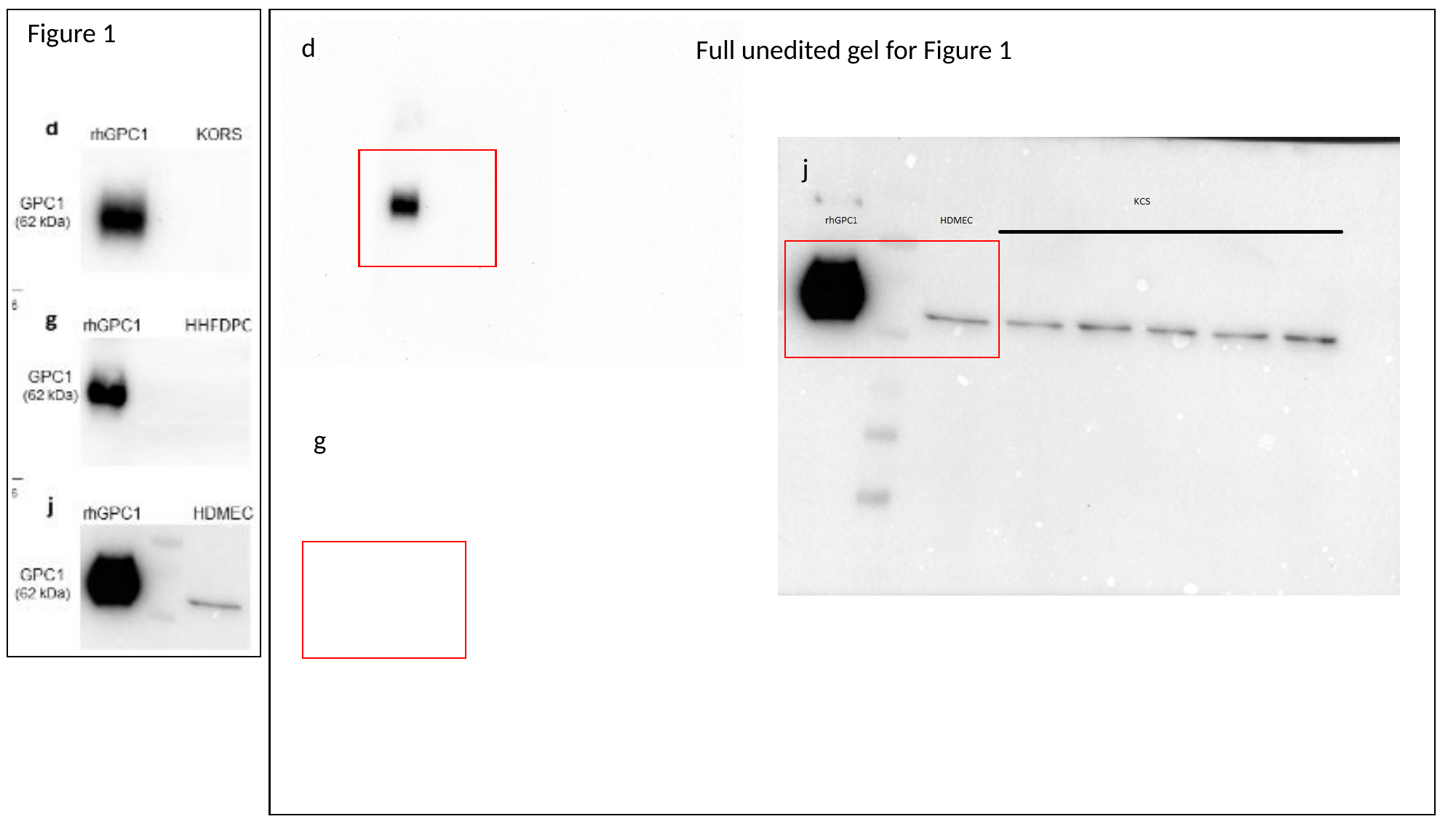

Figure 1
d
Full unedited gel for Figure 1
j
g

## Slide 2
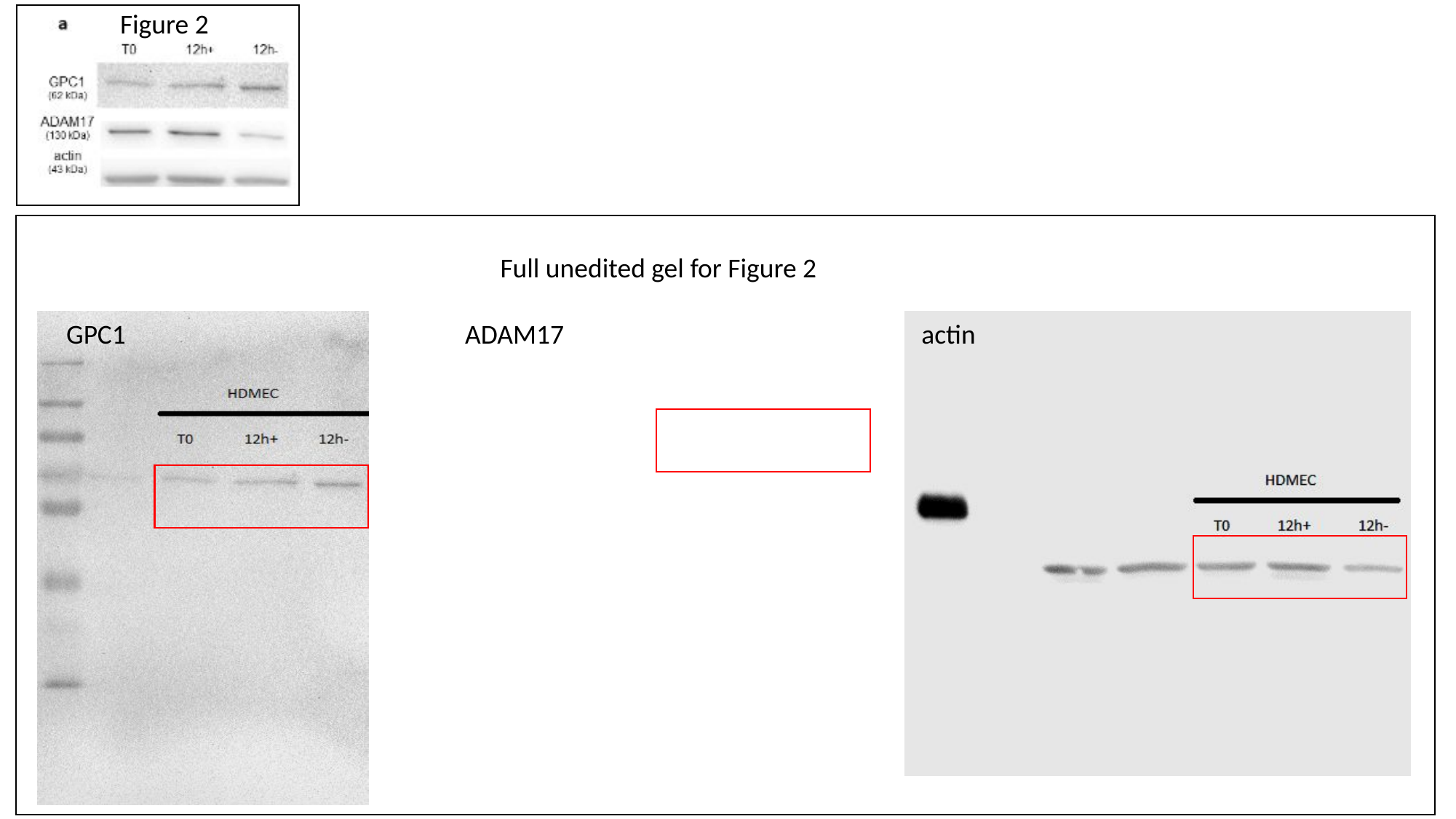

Figure 2
Full unedited gel for Figure 2
GPC1
ADAM17
actin

## Slide 3
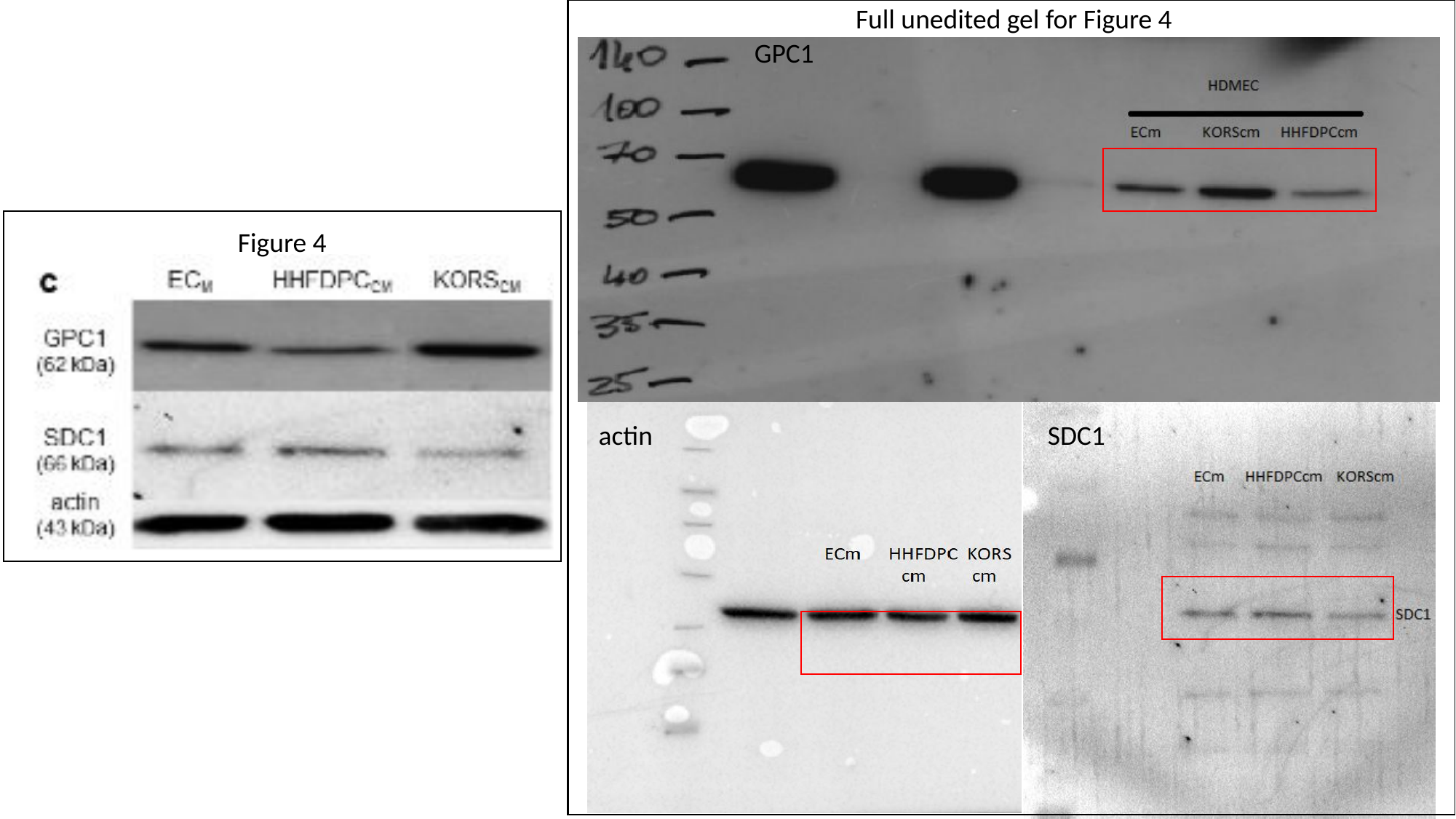

Full unedited gel for Figure 4
GPC1
Figure 4
actin
SDC1

## Slide 4
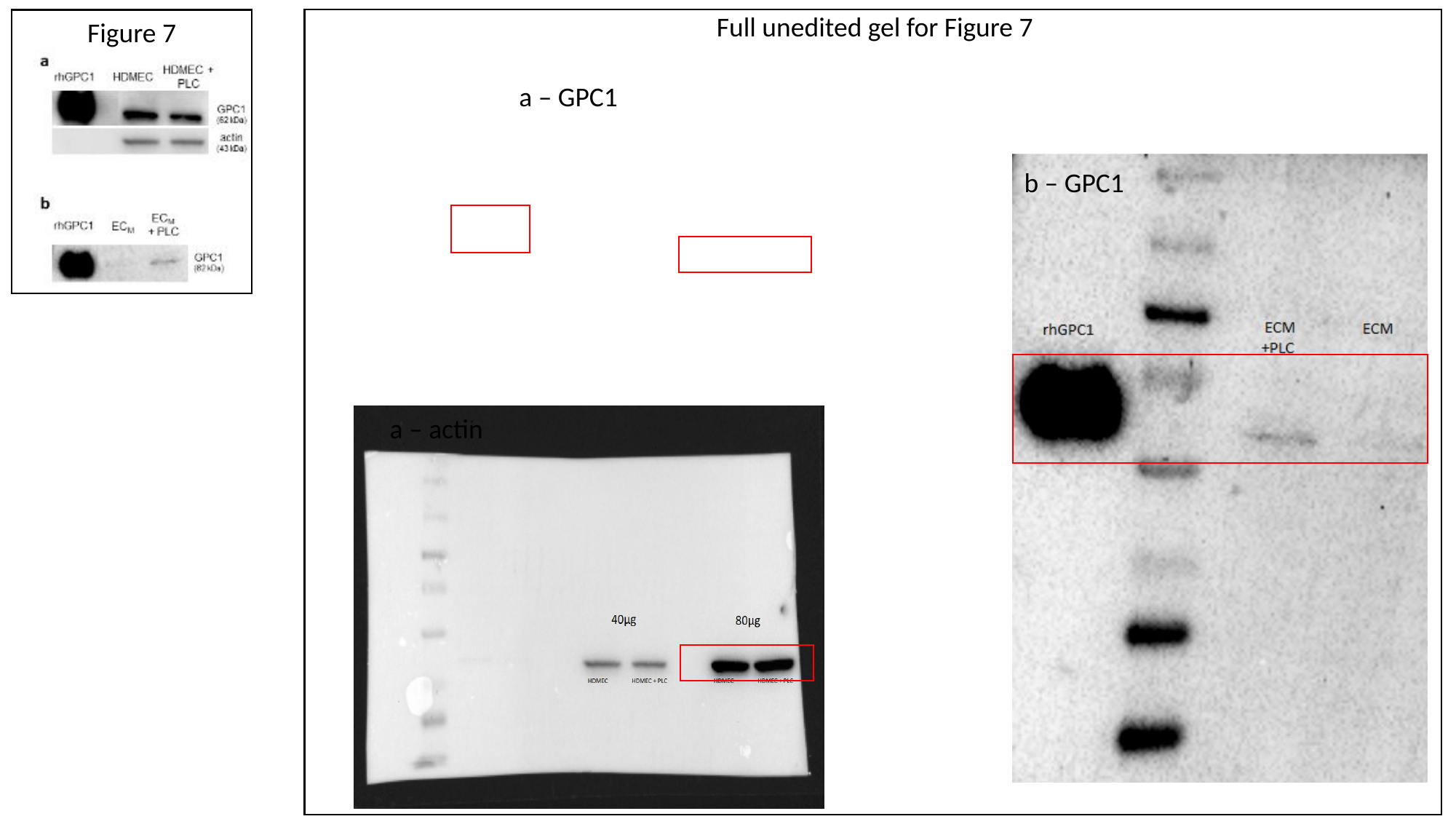

Full unedited gel for Figure 7
Figure 7
a – GPC1
b – GPC1
a – actin

## Slide 5
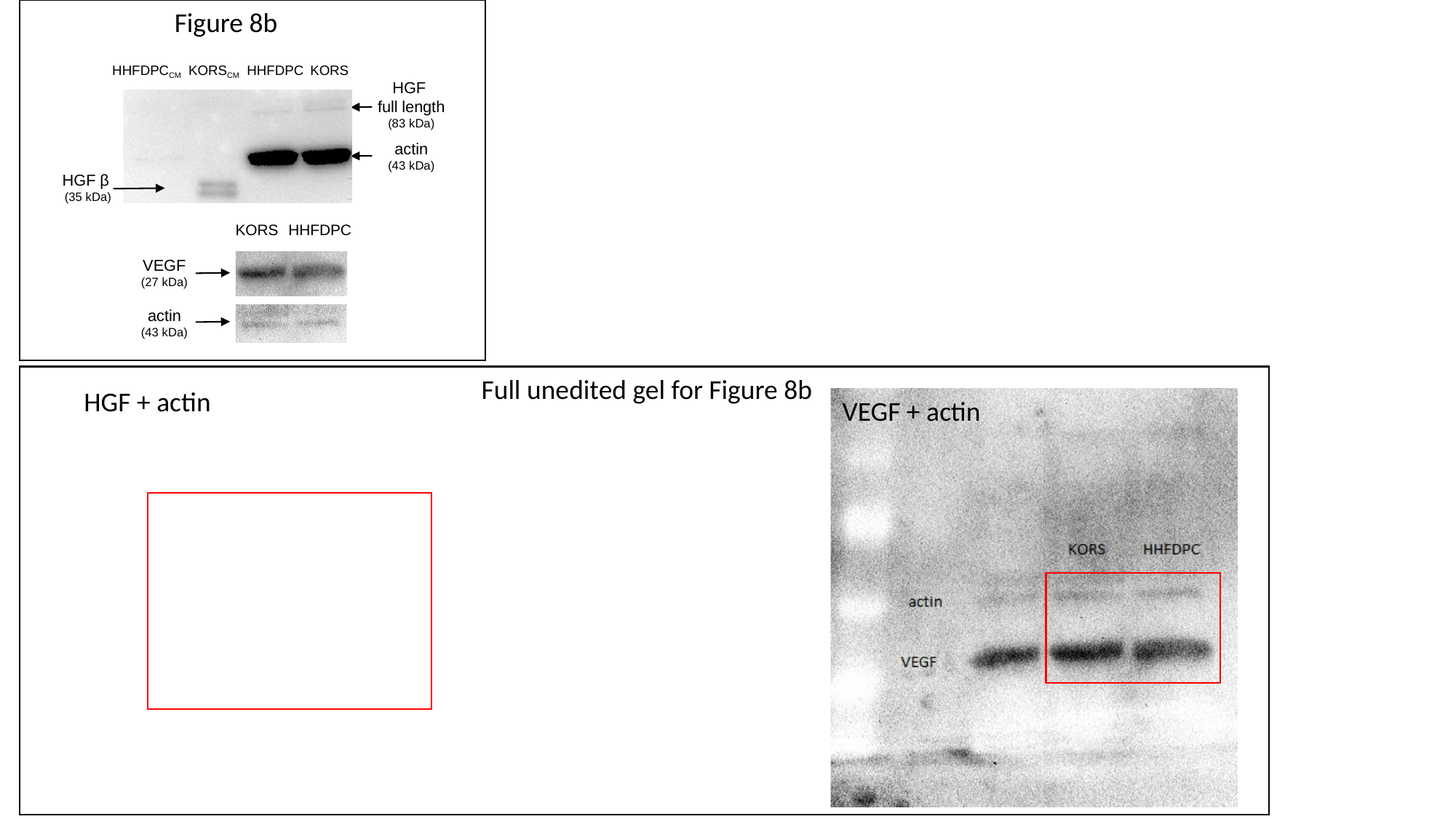

Figure 8b
HHFDPCCM
KORSCM
HHFDPC
KORS
HGF
full length
(83 kDa)
actin
(43 kDa)
HGF β
(35 kDa)
KORS
HHFDPC
VEGF
(27 kDa)
actin
(43 kDa)
Full unedited gel for Figure 8b
HGF + actin
VEGF + actin

## Slide 6
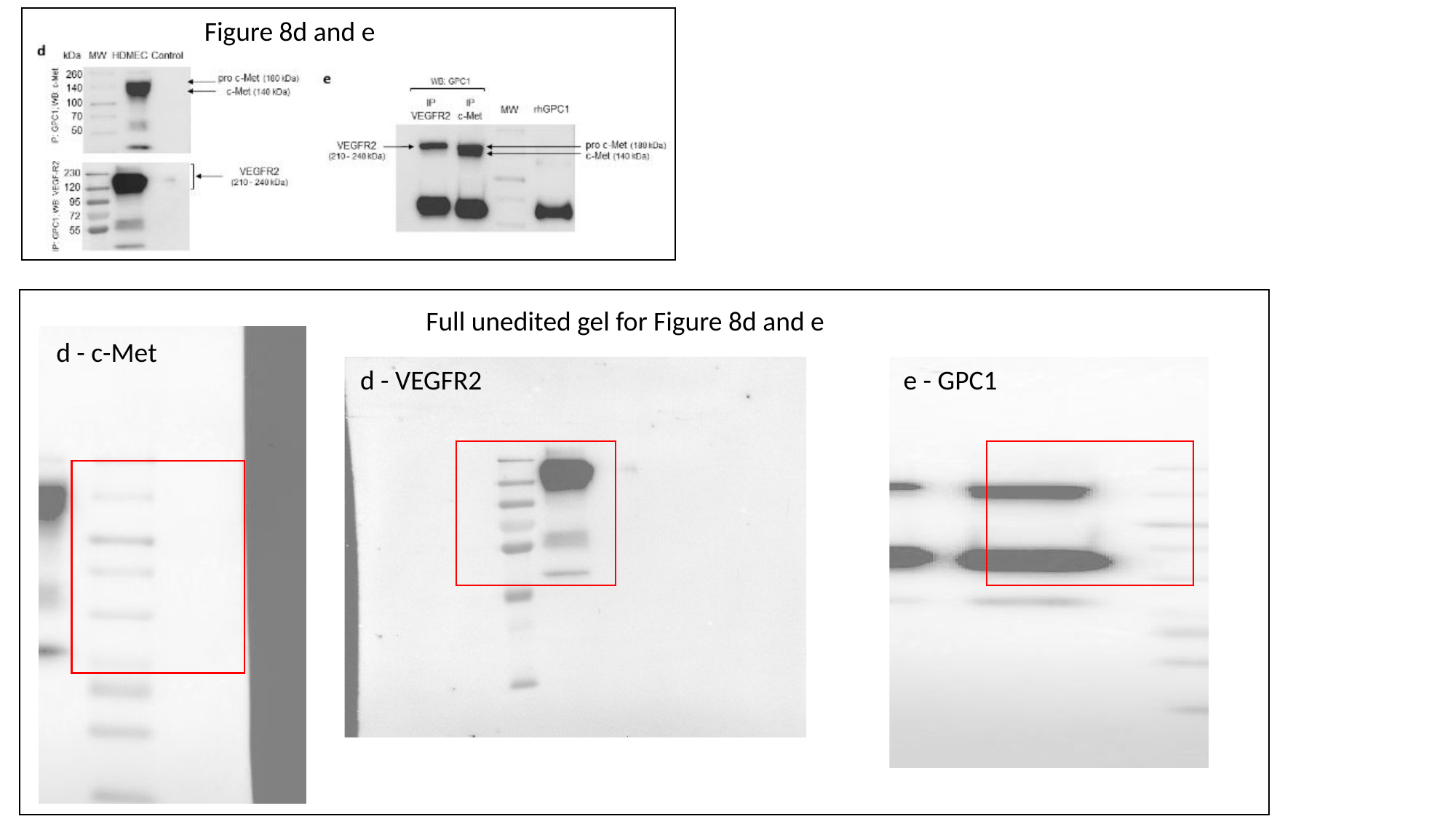

Figure 8d and e
Full unedited gel for Figure 8d and e
d - c-Met
d - VEGFR2
e - GPC1

## Slide 7
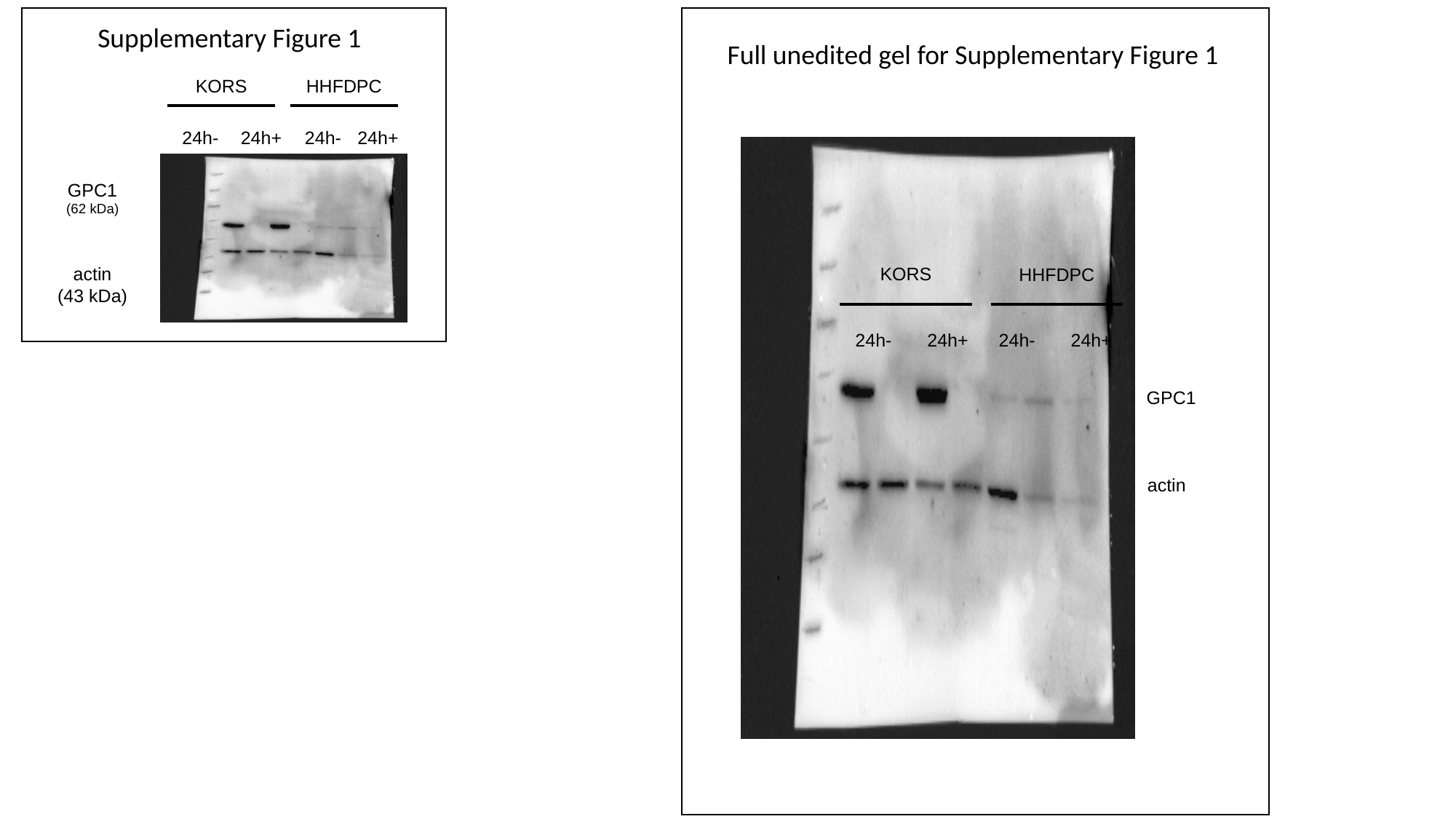

Supplementary Figure 1
Full unedited gel for Supplementary Figure 1
KORS
HHFDPC
24h-
24h+
24h-
24h+
GPC1
(62 kDa)
actin
(43 kDa)
KORS
HHFDPC
24h-
24h+
24h-
24h+
GPC1
actin
