# Supplementary figures and images for: The Glypican-1/HGF/C-Met and Glypican-1/VEGF/VEGFR2 Ternary Complexes Regulate Hair Follicle Angiogenesis
Source: Front Cell Dev Biol. 2021 Dec 8;9:781172. doi: 10.3389/fcell.2021.781172 (PMC8692797; doi:10.3389/fcell.2021.781172)

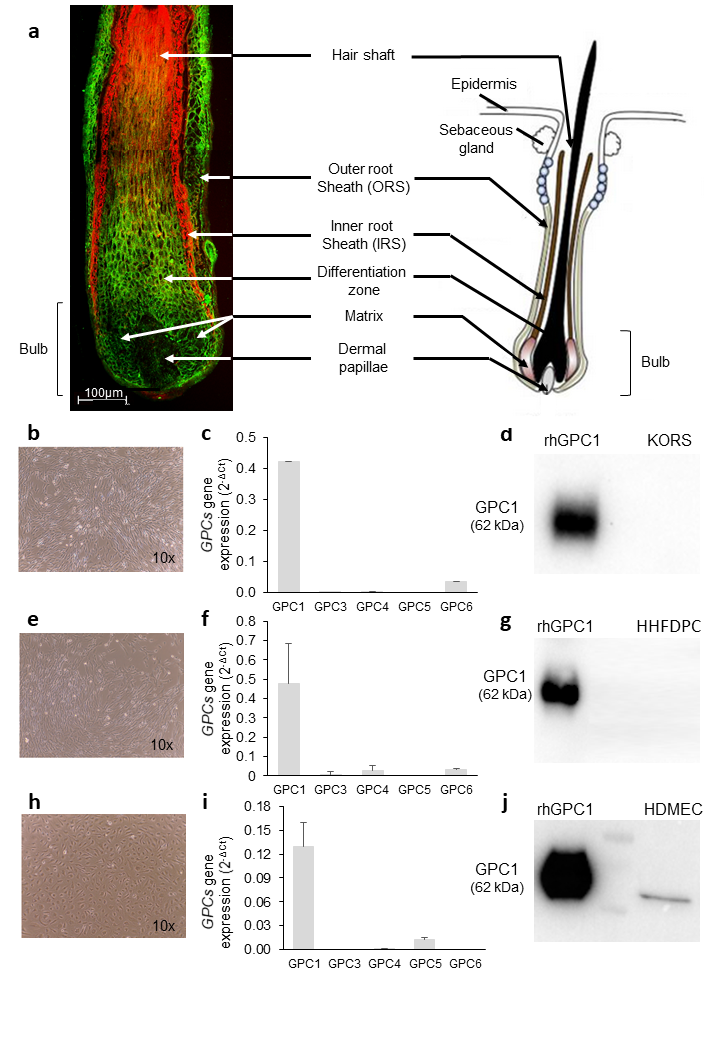

Supplement: Supplementary file 2 [file DataSheet1.zip › Figure 1.TIF]

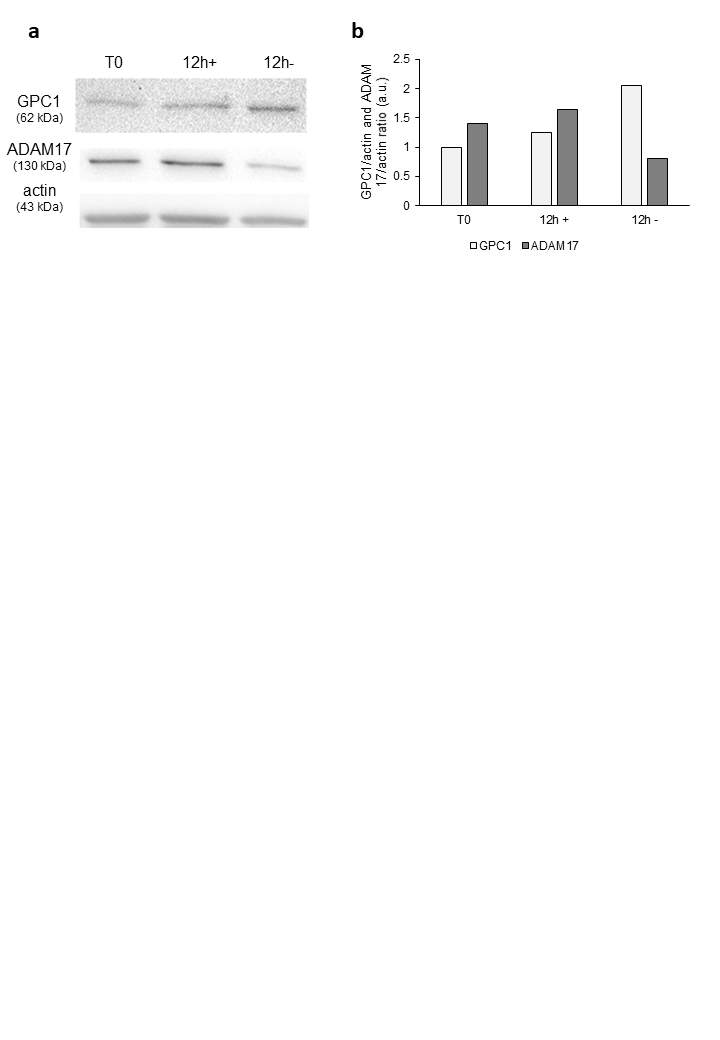

Supplement: Supplementary file 2 [file DataSheet1.zip › Figure 2.TIF]

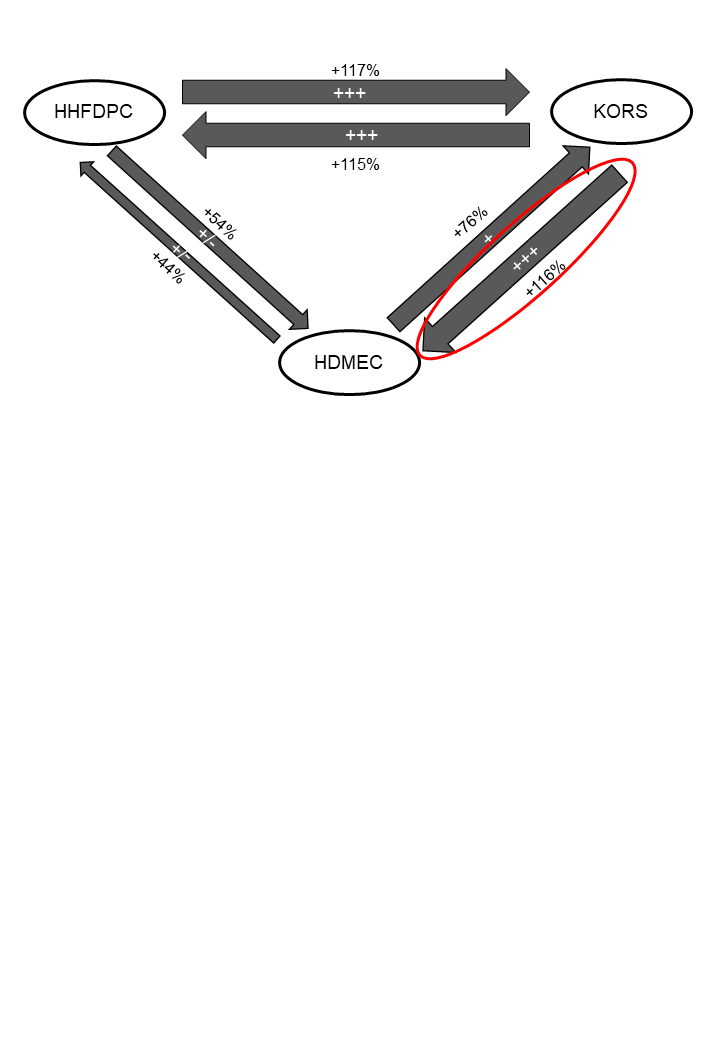

Supplement: Supplementary file 2 [file DataSheet1.zip › Figure 3.TIF]

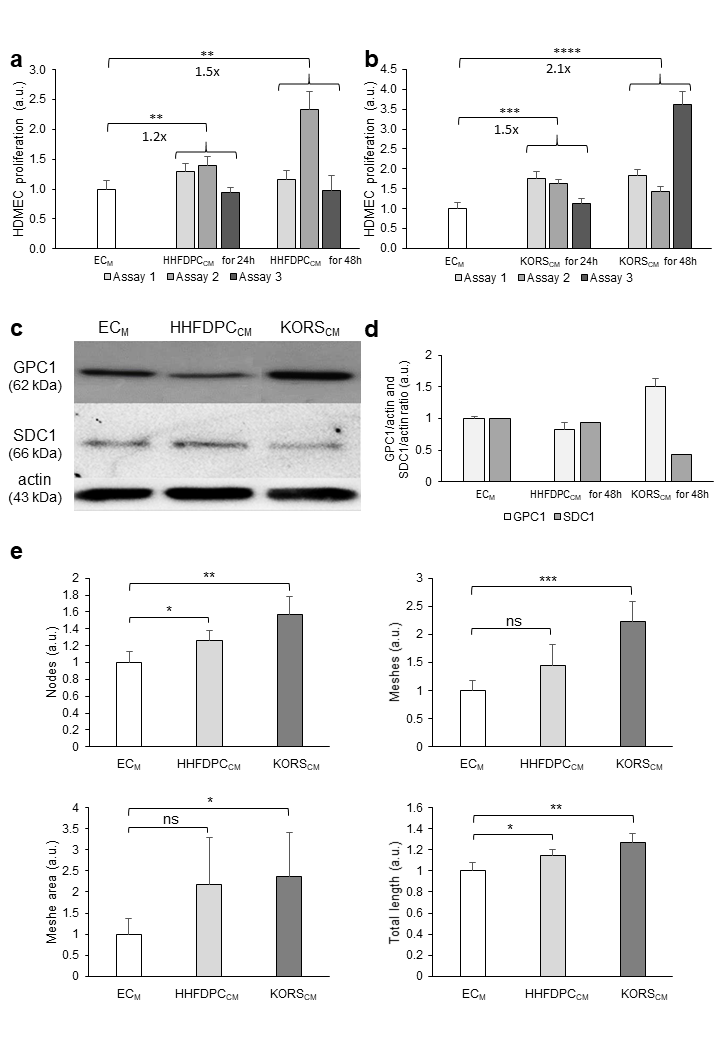

Supplement: Supplementary file 2 [file DataSheet1.zip › Figure 4.TIF]

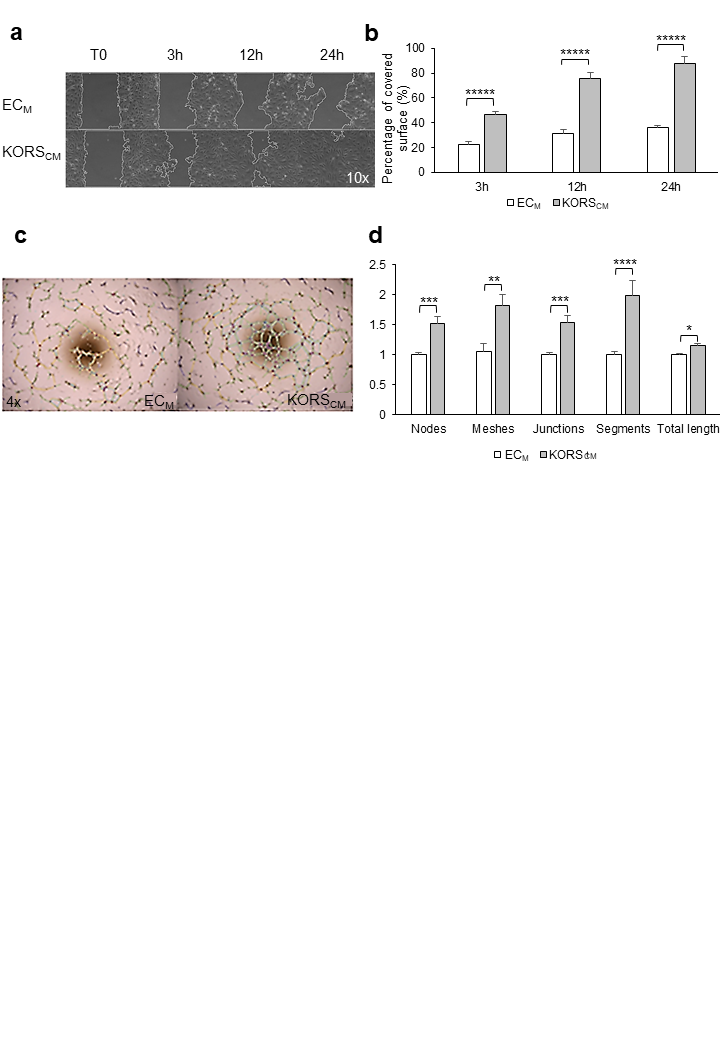

Supplement: Supplementary file 2 [file DataSheet1.zip › Figure 5.TIF]

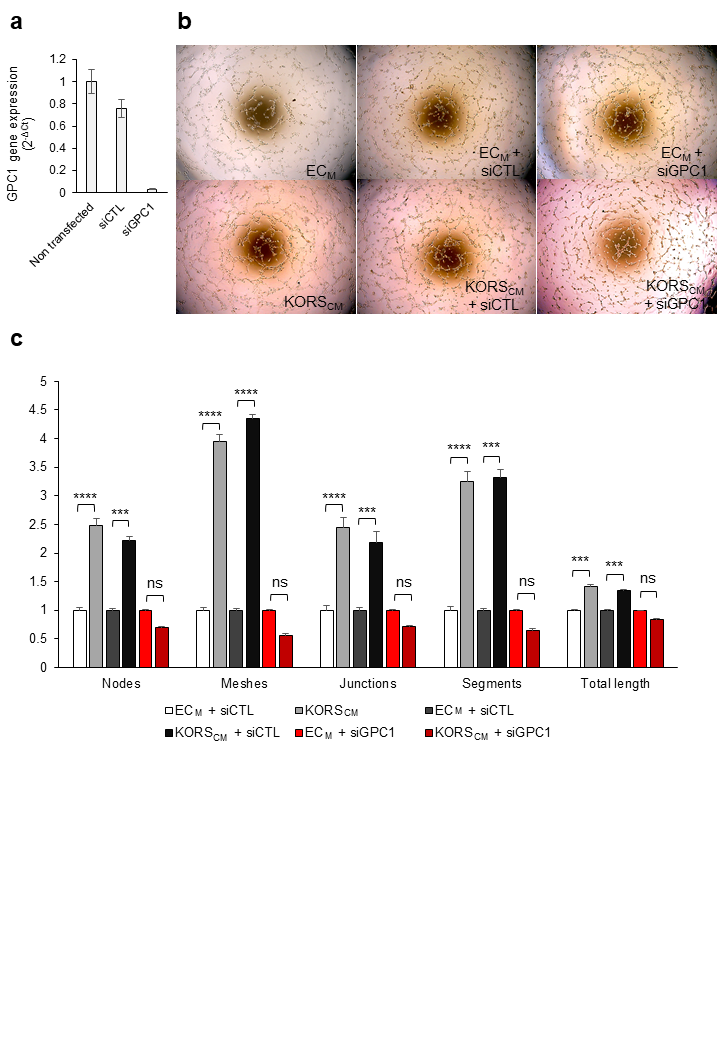

Supplement: Supplementary file 2 [file DataSheet1.zip › Figure 6.TIF]

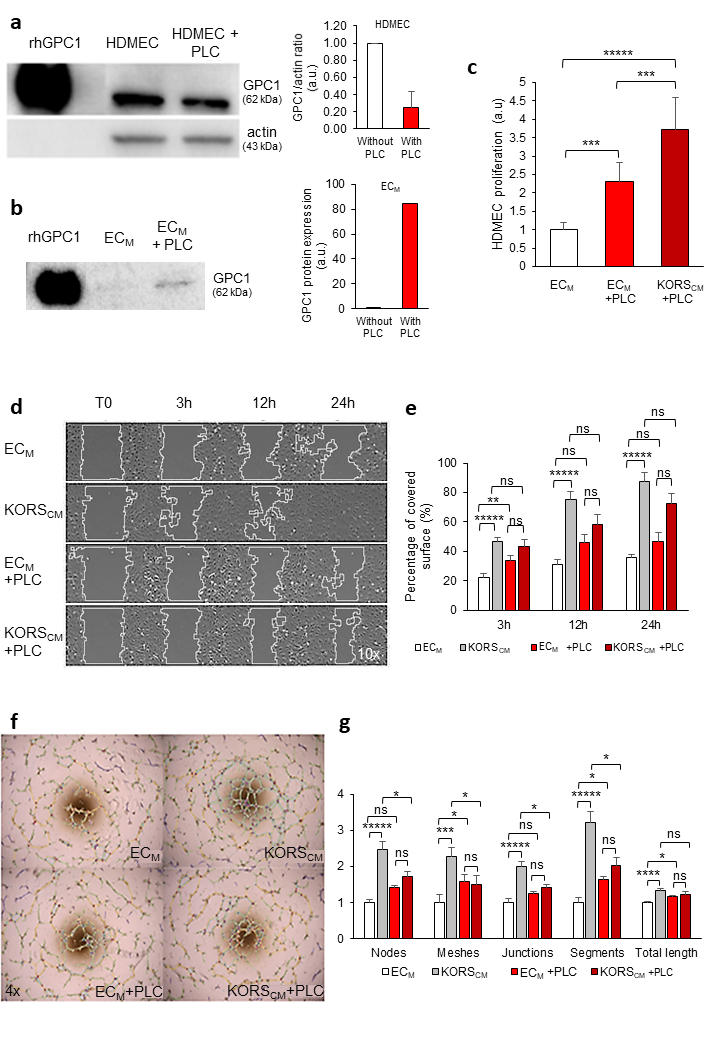

Supplement: Supplementary file 2 [file DataSheet1.zip › Figure 7.TIF]

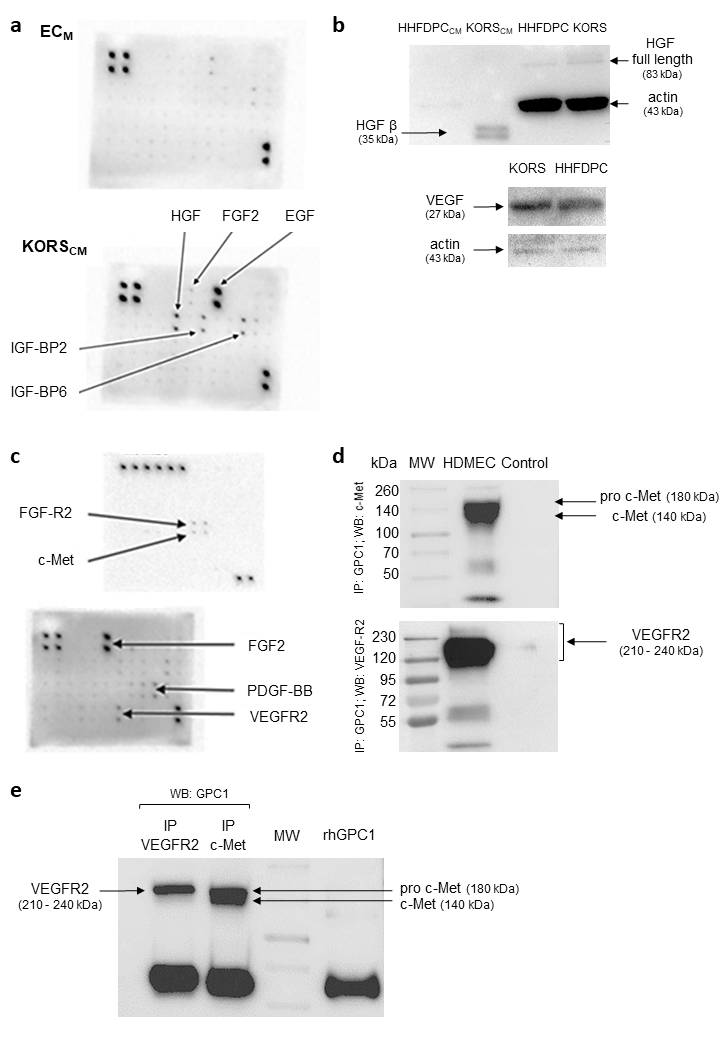

Supplement: Supplementary file 2 [file DataSheet1.zip › Figure 8.TIF]

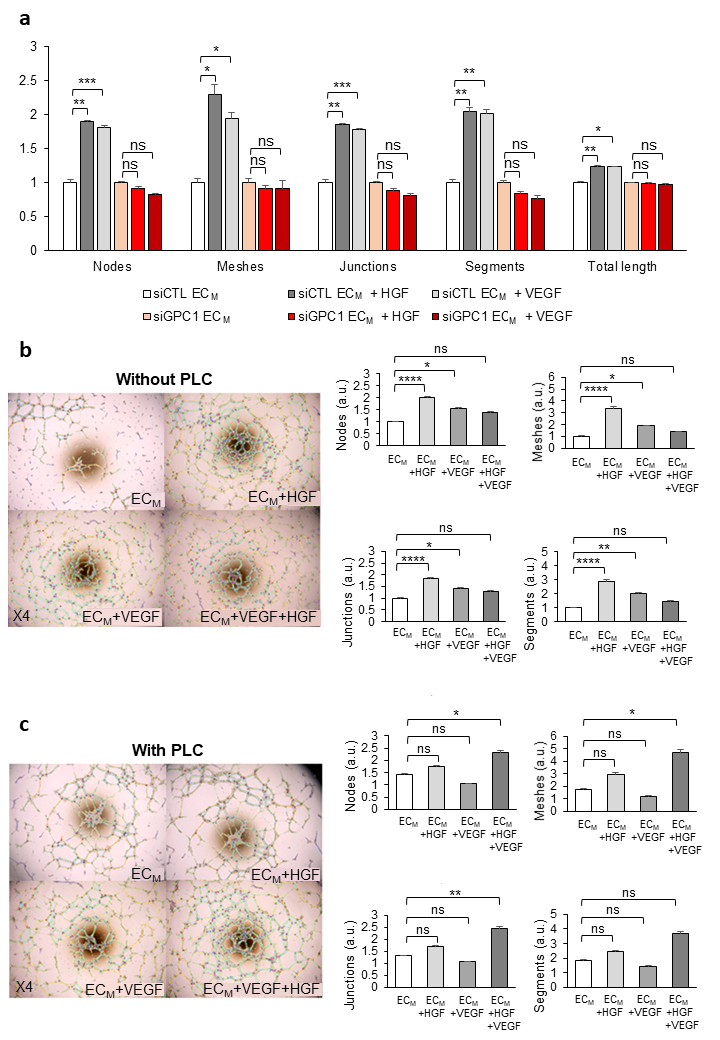

Supplement: Supplementary file 2 [file DataSheet1.zip › Figure 9.TIF]

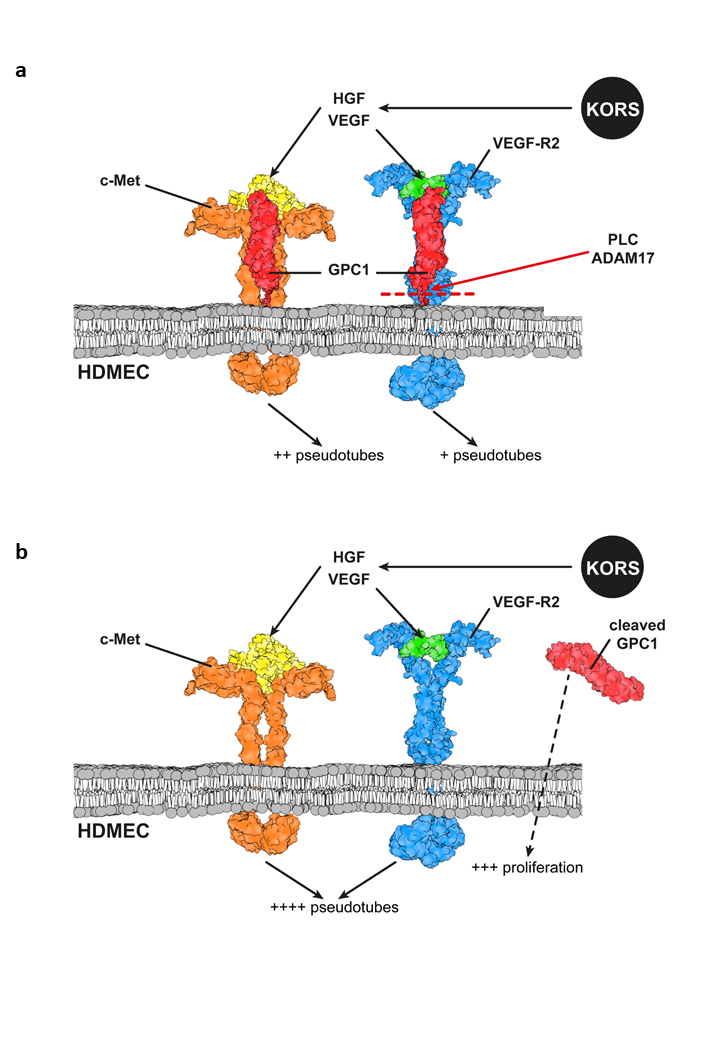

Supplement: Supplementary file 2 [file DataSheet1.zip › Figure 10.TIF]

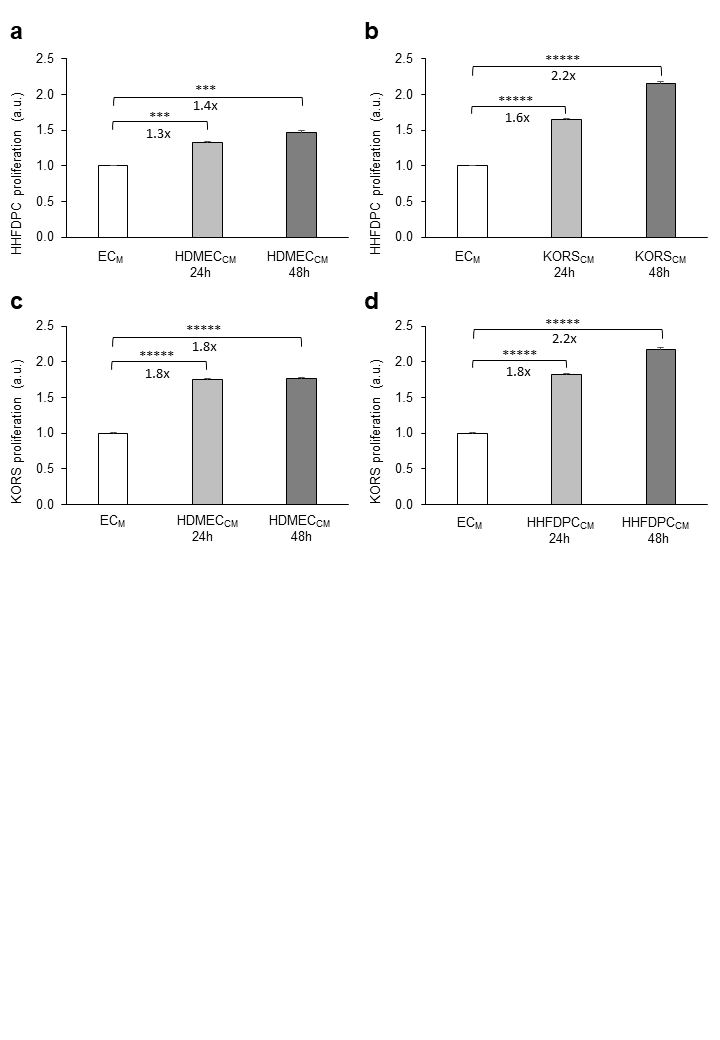

Supplement: Supplementary file 3 [file Image2.TIF]

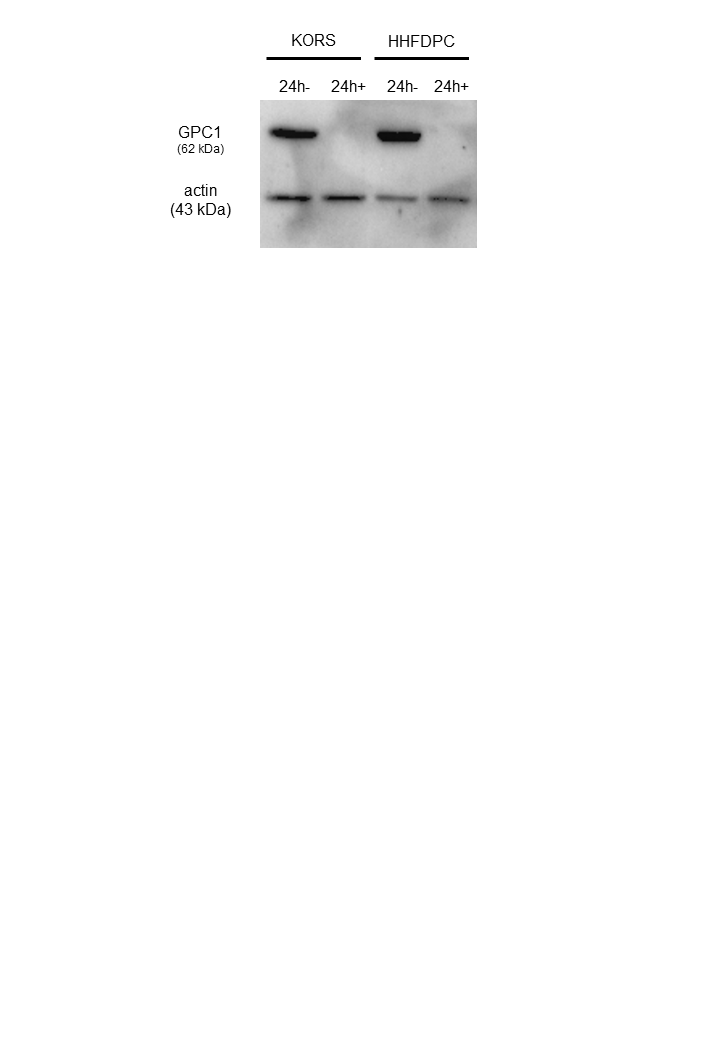

Supplement: Supplementary file 4 [file Image1.TIF]
